# RISC in Entamoeba histolytica: Identification of a Protein-Protein Interaction Network for the RNA Interference Pathway in a Deep-Branching Eukaryote

**DOI:** 10.1128/mBio.01540-21

**Published:** 2021-09-07

**Authors:** Hanbang Zhang, Juliana Veira, Sarah Twitty Bauer, Christopher Yip, Upinder Singh

**Affiliations:** a Division of Infectious Diseases, Department of Internal Medicine, Stanford University School of Medicine, Stanford, California, USA; b Department of Microbiology and Immunology, Stanford University School of Medicine, Stanford, California, USA; University of California Los Angeles

**Keywords:** RNAi, Argonaute, RISC, mass spectrometry, parasite, RNA interference, parasitology

## Abstract

Entamoeba histolytica is a protozoan parasite that causes amebiasis in humans and is a major health concern in developing countries. Our previous work revealed a functional RNA interference (RNAi) pathway in *Entamoeba*. Several unusual features encompass the RNAi pathway in the parasite, including small RNAs (sRNAs) with a 5′-polyphosphate structure (identified to date only in *Entamoeba* and nematodes) and the conspicuous absence of a canonical Dicer enzyme. Currently, little is known about the *Entamoeba* RNA-induced silencing complex (RISC), which is critical in understanding how RNAi is achieved in the parasite. In this study, we examined the RISC of *Eh*Ago2-2, the most highly expressed Argonaute protein in *Entamoeba*. We identified 43 protein components of *Eh*Ago2-2 RISC with a broad range of functional activities. Two proteins with nucleosome assembly protein (NAP) domains, not previously observed in other RNAi systems, were identified as novel core members of amebic RISC. We further demonstrated the interaction of these NAPs with Ago using an *in vitro* recombinant system. Finally, we characterized the interaction network of five RISC components identified in this study to further elucidate the interactions of these RNAi pathway proteins. Our data suggest the presence of closely interacting protein groups within RISC and allowed us to build a map of protein-protein interactions in relation to Ago. Our work is the first to elucidate RISC components in *Entamoeba* and expands the current knowledge of RISC to a deep-branching single-celled eukaryote.

## INTRODUCTION

RNA interference (RNAi) and its gene silencing mechanism are evolutionally conserved among most eukaryotes, ranging from single-celled protists to humans (1–4). RNAi can control many important biological processes such as antiviral defense, chromatin and transposon silencing, germ line differentiation, and posttranscriptional and transcriptional gene silencing (PTGS and TGS, respectively) (5–8). In all known RNAi systems, Argonaute (Ago) proteins and small RNAs (sRNAs) form a ribonucleoprotein complex along with many other partner proteins, collectively known as the RNA-induced silencing complex (RISC) (9–11).

RISCs in model systems such as yeast, *Drosophila*, and mammals have been studied extensively in the past few decades (12–15). The RISC components of the small interfering RNA (siRNA) or microRNA (miRNA) PTGS pathway contain several highly conserved proteins, such as Dicer, double-stranded RNA-binding domain (dsRBD) protein, DEXD-box RNA helicase, and RNA binding proteins (13, 16). In contrast, the RISC involved in the TGS pathway often contains chromatin-interacting proteins such as TAS3 (DNA chromatin remodeling proteins) and CHP1 (the chromodomain protein in yeast *Pombe*) (15, 17). The Piwi RISC includes chromatin-associated protein HP-1, RecQ1, Tudor staphylococcal nuclease (TSN), and protein R-methyl transferase-5 (PRMT5) (14, 18, 19). Thus, the RISC content is tightly linked to Ago type and the RNAi gene-silencing mechanism.

RNAi pathway proteins have previously been identified in several pathogenic protozoan parasites such as Trypanosoma brucei, Toxoplasma gondii, Giardia lamblia, Trichomonas vaginalis, and Entamoeba histolytica (20, 21). Functional evidence of RNAi-based silencing has been further demonstrated in T. brucei, T. gondii, and E. histolytica, while it is still in question for G. lamblia and T. vaginalis (22). There is limited knowledge on Ago RISC in parasitic protozoa, with only one published study on *Tg*Ago RISC (23). The T. gondii RISC content is similar to human Ago2 RISC components, as the two systems share many orthologs, including RNA helicases, RNA binding proteins, and transcriptional and translational proteins (23).

E. histolytica causes amoebiasis in humans, resulting in approximately 100,000 deaths annually worldwide (24, 25). Common symptoms of E. histolytica infections include dysentery, colitis, and liver abscesses (26, 27). The pathogenesis of parasite infection is closely linked to its gene regulation mechanism. We previously demonstrated that E. histolytica possesses active RNAi machinery, and all three *Eh*Ago proteins (*Eh*Ago2-1, -2-2, -2-3 as EHI_186850, EHI_125650, and EHI_177170, respectively) associate with sRNA populations (28–30). *Eh*Ago2-2 is the most highly expressed Ago protein in *Entamoeba*. *Eh*Ago2-2 localizes primarily to the nucleus and mediates transcriptional gene silencing via 27-nucleotide (nt) secondary small RNAs (31, 32). However, many facets of RNAi still remain to be determined in this nonmodel organism. First, the E. histolytica genome appears to lack a canonical Dicer enzyme on the basis of homology searches. The Dicer enzyme is an important protein involved in the RNAi pathway that processes double-stranded RNA (dsRNA) into siRNAs. Canonical Dicer enzymes found in plants and animals contain N-helicase, PAZ, and dsRBD domains and two RNase III domains. However, a noncanonical Dicer enzyme found in budding yeast only has RNase III and dsRBD domains. We identified a protein with a single RNase III domain (EHI_068740) which appears to have weak dsRNA processing activity (33, 34). Whether this RNase III domain protein interacts with *Eh*Ago proteins remains to be determined. Second, E. histolytica sRNA populations have a 5′-polyphosphate structure (28), indicating these sRNAs are likely products of RNA-dependent RNA polymerase (RdRP) (35). The connections between *Eh*RdRP and *Eh*Ago complexes have not yet been studied in the parasite. Finally, the RISC components associated with each *Eh*Ago protein have not been completely characterized or studied.

Here, we report a comprehensive analysis of *Eh*Ago2-2 RISC. We identified 43 proteins that interact with *Eh*Ago2-2. These 43 proteins are annotated with diverse functions ranging from transcriptional and translation regulation, sRNA loading, RNA binding, and vesicle trafficking to protein folding and modification and may be involved in cell signaling and various metabolic pathways. Using two *Eh*Ago2-2 functional mutants, which either abolish sRNA binding or affect *Eh*Ago2-2 localization, we showed the dynamic Ago association patterns of the RISC components and identified a stable core of 23 proteins. Among the core RISC components are two proteins with a nucleosome assembly protein (NAP) domain. We demonstrated a specific interaction between the two NAPs and Ago using an *in vitro* recombinant system. Interestingly, NAPs have not been previously reported in other Ago RISC systems. Furthermore, we identified protein components associated with two *Eh*RdRPs and found that more than half of the Ago RISC components are shared with them, suggesting that these proteins may function together to orchestrate and effectively carry out RNAi in the parasite. Finally, we characterized five RISC components and demonstrated the existence of closely bound protein groups within the RISC and built a preliminary protein-protein interaction network in relation to *Eh*Ago2-2. Our work is the first to elucidate Ago RISC cofactors in *Entamoeba*, and the findings expand current knowledge on RISC to a deep-branching single-celled eukaryote.

## RESULTS

### Immunoprecipitation of Myc-tagged Ago protein.

To identify RISC components associated with *Eh*Ago2-2, we relied on three previously constructed cell lines: Myc-*Eh*Ago2-2, Myc-*Eh*Ago2-2^ΔNLS-DR^, and Myc-DHFR-*Eh*Ago2-2^PAZ-mut^. All three cell lines overexpress an N-terminal Myc-tagged Ago protein (wild type [WT] or mutants). As noted in reference 30, Myc-tagged *Eh*Ago2-2 exhibits strong binding to sRNA and localizes primarily to the nucleus, with a faint cytoplasmic signal. The Myc-tagged *Eh*Ago2-2^ΔNLS-DR^ variant exhibits sRNA binding activity but does not localize to the nucleus. Under similar assay conditions, the Myc-tagged DHFR-*Eh*Ago2-2^PAZ-mut^ variant has a localization pattern similar to that of WT *Eh*Ago2-2 but is impaired in its ability to bind sRNA. Table 1 summarizes the observed features of *Eh*Ago2-2 for these three cell lines. To identify RISC components specifically associated with *Eh*Ago2-2, we included a cell line that overexpresses an irrelevant protein (luciferase without a Myc tag) to serve as a drug selection control. We prepared whole-cell lysates from the four cell lines and performed anti-Myc immunoprecipitation (IP) under the same conditions. Western blotting and silver staining were used to check protein enrichment following IP (examples of *Eh*Ago2-2 WT and the ΔNLS-DR mutant IPs are shown in Fig. S1 in the supplemental material). We observed a specific pulldown of Myc-tagged Ago protein in the IP sample; actin showed no signal in these IPs, indicating the specific enrichment of the Myc-tagged Ago in the experiment. We performed three biological replicates of IP samples for each cell line. Proteins that were pulled down by IP were released from the beads using SDS-PAGE sample loading buffer, separated by SDS-PAGE, and sent for liquid chromatography-tandem mass spectrometry (LC-MS/MS) analyses after in-gel trypsin digestion.

**TABLE 1:** Cell lines used for Ago RISC analysis

Cell lines	Description	Ago	Ago localization	sRNA binding
Myc-*Eh*Ago2-2	N-Myc-tagged *Eh*Ago2-2 protein	Wild type	enriched in nucleus	Yes
Myc-*Eh*Ago2-2^ΔNLS-DR^	N-Myc-tagged *Eh*Ago2-2 mutant (ΔNLS-DR)	aa^*a*^ 761–937 deletion	mostly in cytoplasm	Yes
Myc-DHFR-*Eh*Ago2-2^PAZ-mut^	N-Myc-tagged *Eh*Ago2-2 mutant (PAZ-mut) by protein destabilization domain approach	aa Y267A, Y268A	enriched in nucleus	No
Luc control	Luciferase protein without Myc-tag	NA^*b*^	NA	NA

a aa, amino acid.

b NA, not applicable.

10.1128/mBio.01540-21.1 FIG S1 Specific pulldown of *Eh*Ago2-2 and its mutants by anti-Myc IP. Western blotting and silver staining were used to check anti-Myc IP samples prior to mass spectrometry analyses. Shown are examples of *Eh*Ago2-2 and *Eh*Ago2-2^ΔNLS-DR^ IPs. Two identical SDS-PAGE gels were loaded as indicated (input lysate, IP supernatant, and IP bead elution). (Top) Anti-Myc Western blot shows Myc signal in the IP elution lanes for *Eh*Ago2-2 and *Eh*Ago2-2^ΔNLS-DR^ but not the control, showing specific pulldown of Myc-tagged protein. (Middle) Anti-actin Western shows that actin is almost depleted in the IP elution lanes compared to that in lanes for input lysate and IP supernatant. (Bottom) Silver stain shows that IP elution samples have lost most protein bands present in the input. The thick band at 50 kDa is IgG from the beads. Arrow indicates the detected protein band for *Eh*Ago2-2 and *Eh*Ago2-2^ΔNLS-DR^ in IP elution samples. Download FIG S1, PDF file, 0.5 MB. Copyright © 2021 Zhang et al. 2021 Zhang et al. https://creativecommons.org/licenses/by/4.0/ This content is distributed under the terms of the Creative Commons Attribution 4.0 International license.

### Selection criteria and RISC content analysis for *Eh*Ago2-2.

**FIG 1:**
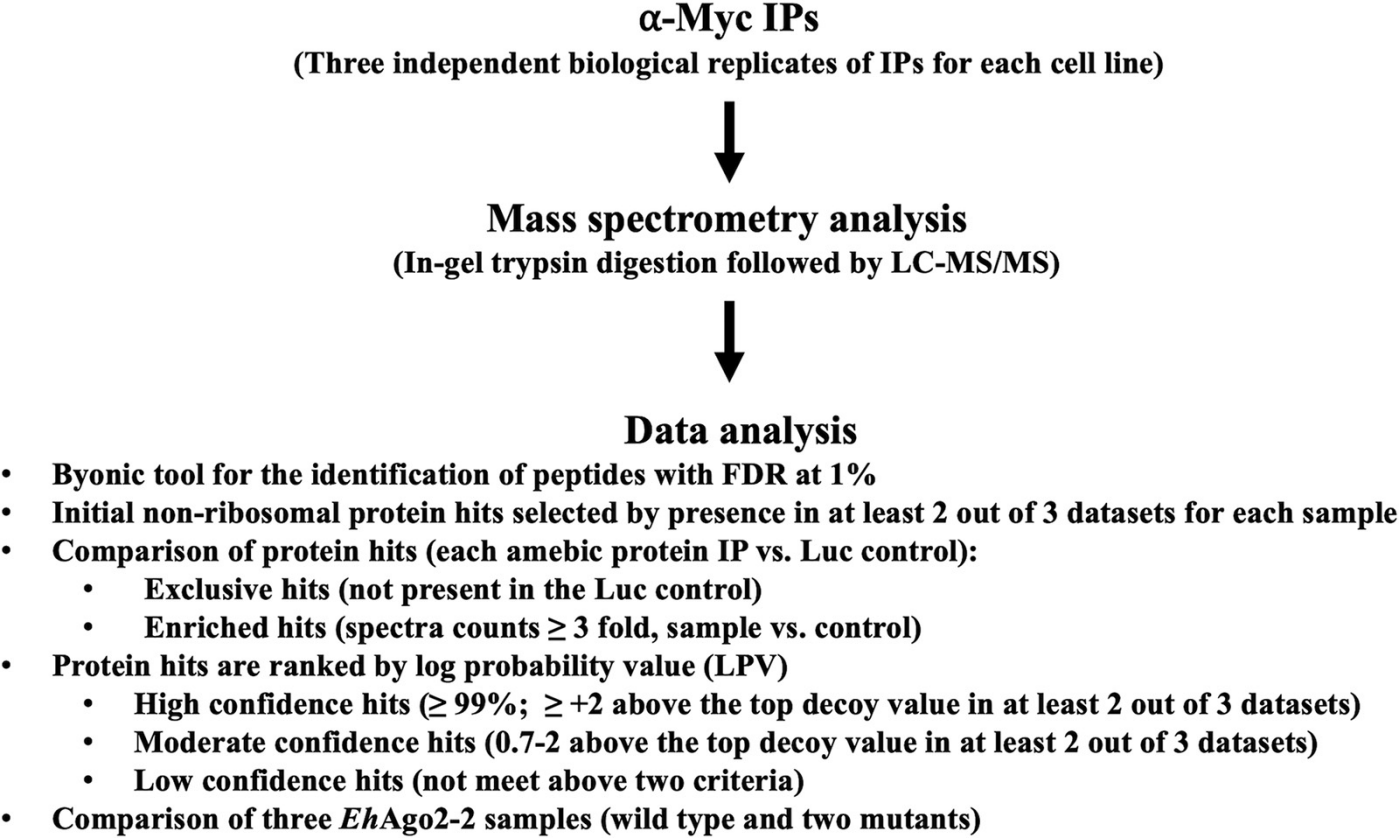
Schematic of experimental design and mass spectrometry analysis and its data processing criteria in this study. Each IP sample, performed in triplicates, was processed by in-gel trypsin digestion for LC-MS/MS. Raw data were analyzed by the Byonic tool for the identification of protein hits. Stepwise selection criteria, performed as listed, were used to prioritize protein hits.

Proteomic analysis of interaction networks often has variability or reproducibility issues, as many factors/steps are involved during sample preparation and instrumental detection. To limit potential false positives, we used the following experimental parameters to determine the protein candidates (shown in Fig. 1): Byonic software analysis was performed on mass spectrometry raw data with a false-discovery rate (FDR) of 1% to report peptides (36) and inferred proteins from the E. histolytica genome. Each mass spectrometry run typically generated 100 to 150 protein hits for each IP sample. We first separated nonribosomal protein hits from ribosomal hits. Nonribosomal protein hits that appeared in at least 2 of 3 data sets were selected for further analysis. We then compared either the WT Ago or Ago mutants with the Luc control and generated two sublists thereafter: (i) an exclusive list, consisting of protein hits that are in the Ago samples but not in the control, and (ii) an enriched list, consisting of protein hits that overlapped between the Ago sample and the control but with ≥ 3-fold enrichment of protein hits in the Ago samples based on average spectrum counts. The data analysis workflow for WT *Eh*Ago2-2 is shown in Fig. S2A. There were 61 nonribosomal protein hits detected in at least 2 of the 3 data sets, while the luciferase (Luc) control had 27 nonribosomal protein hits under the same criteria. A comparison of *Eh*Ago2-2 and the Luc control resulted in 41 exclusive protein hits and two enriched protein hits based on the criteria established as described above. Thus, we identified 43 protein hits as components of WT *Eh*Ago2-2 RISC.

10.1128/mBio.01540-21.2 FIG S2 Identification of protein hits by Venn diagram analyses for *Eh*Ago2-2 and its mutants. For each reported Byonic data set, ribosomal hits were separated from nonribosomal protein hits. Analysis continued only with nonribosomal protein hits. We used three replicates for each cell line to identify initial protein hits present in at least 2 of 3 data sets. *Eh*Ago2-2 or mutants were compared with the Luc control, and two protein lists were generated: an exclusive list and an enriched list. (A) Venn diagram analysis of *Eh*Ago2-2 identifies 41 exclusive protein hits and 2 enriched protein hits. (B) Venn diagram analysis for *Eh*Ago2-2^ΔNLS-DR^ identifies 32 exclusive protein hits and 1 enriched protein hit. (C) Venn diagram analysis for *Eh*Ago2-2^PAZ-mut^ identifies 98 exclusive protein hits and 6 enriched protein hits. Download FIG S2, PDF file, 1.6 MB. Copyright © 2021 Zhang et al. 2021 Zhang et al. https://creativecommons.org/licenses/by/4.0/ This content is distributed under the terms of the Creative Commons Attribution 4.0 International license.

The 43 identified protein hits were further weighed based on their Byonic score (|log *P* value| [LPV]). The top decoy value was used to separate each sample data set into three categories: high-confidence hits (≥+2 above the top decoy LPV in at least 2 of 3 data sets, which indicates ≥ 99% confidence level); moderate-confidence hits (above the top decoy LPV and within its +0.7 to 2 range in at least 2 of 3 data sets, which indicates a confidence level of 80% to 99%); and low-confidence hits (does not meet the above-described two criteria). However, it is important to note that low-confidence protein hits can still identify true partner proteins that associate with RISC. Variables such as low protein expression or limited trypsin digestion sites can contribute to how these proteins are detected and ranked by mass spectrometry, thus affecting their representation in our data sets. Table 2 lists the 43 protein hits as components of WT *Eh*Ago2-2 RISC.

**TABLE 2:**
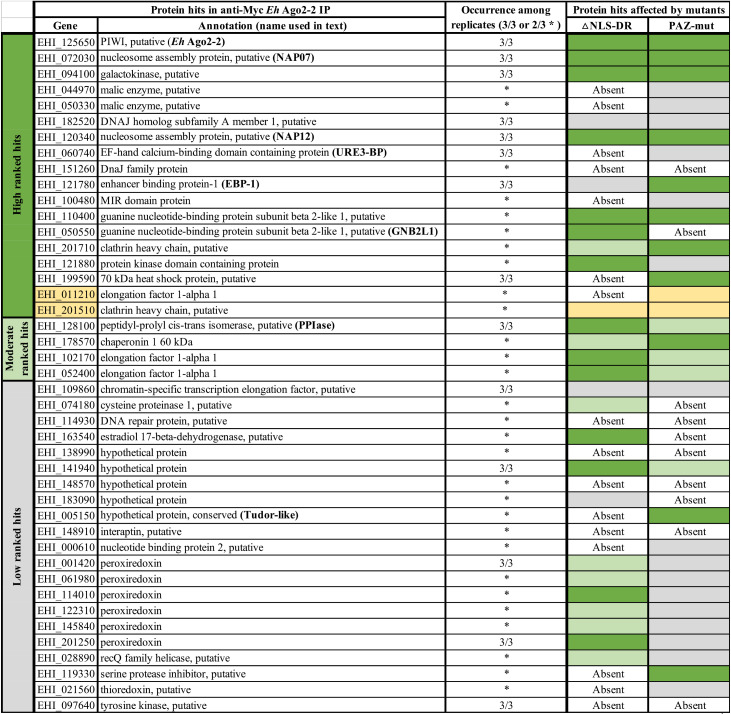
List of *Eh*Ago2-2 RISC components and effects by two functional mutants^*a*^

a Listed are *Eh*Ago2-2 RISC protein hits and their annotations. There are 41 exclusive and 2 enriched (highlighted in light yellow) protein hits identified by mass spectrometry analysis (Fig. S2A in the supplemental material). High-confidence hits are in green (≥2 above top decoy score, with ≥99% confidence level); moderate-confidence hits are in light green (above top decoy score but in the range +0.7 to 2, with confidence level 80% to 99%); low-confidence hits are in gray (hits that do not meet the two criteria). Protein hits identified either by all three data sets (3/3) or 2 of 3 data sets (*) are listed. Our mass spectrometry analyses for two functional *Eh*Ago2-2 mutants (Table S1) show RISC components can be affected by mutants, with some protein hits being absent or showing association level change: either weakening (from green in WT to gray in mutant) or strengthening (from gray in WT to green in mutant) under each mutant condition.

10.1128/mBio.01540-21.8 TABLE S1 List of RISC components for two *Eh*Ago2-2 mutants that were identified by mass spectrometry analysis. (A) List of *Eh*Ago2-2^ΔNLS-DR^ RISC components identified by mass spectrometry analysis. Listed are genes and their annotations for 32 exclusive and 1 enriched (in light yellow) protein hits identified by mass spectrometry analysis (Fig. S2B). (B) List of *Eh*Ago2-2^PAZ-mut^ RISC components identified by mass spectrometry analysis. Listed are genes and their annotations for 98 exclusive and 6 enriched (in light yellow) protein hits identified by mass spectrometry analysis (Fig. S2C). Similarly to those in Table 2, these protein hits were ranked by Byonic score (|log *P* value|): high-ranked hits are in green (≥2 above top decoy protein score, with ≥99% confidence level); moderate-ranked hits are in light green (above top decoy protein score but in the range 0.7 to 2, with confidence level 80% to 99%); and low-ranked hits are in grey (did not meet the other two criteria). Information on protein hits that were identified either by all three data sets (3/3) or 2 of 3 data sets (*) are listed. Download Table S1, XLSX file, 0.02 MB. Copyright © 2021 Zhang et al. 2021 Zhang et al. https://creativecommons.org/licenses/by/4.0/ This content is distributed under the terms of the Creative Commons Attribution 4.0 International license.

### Functional groups represented by *Eh*Ago2-2 RISC components.

**FIG 2:**
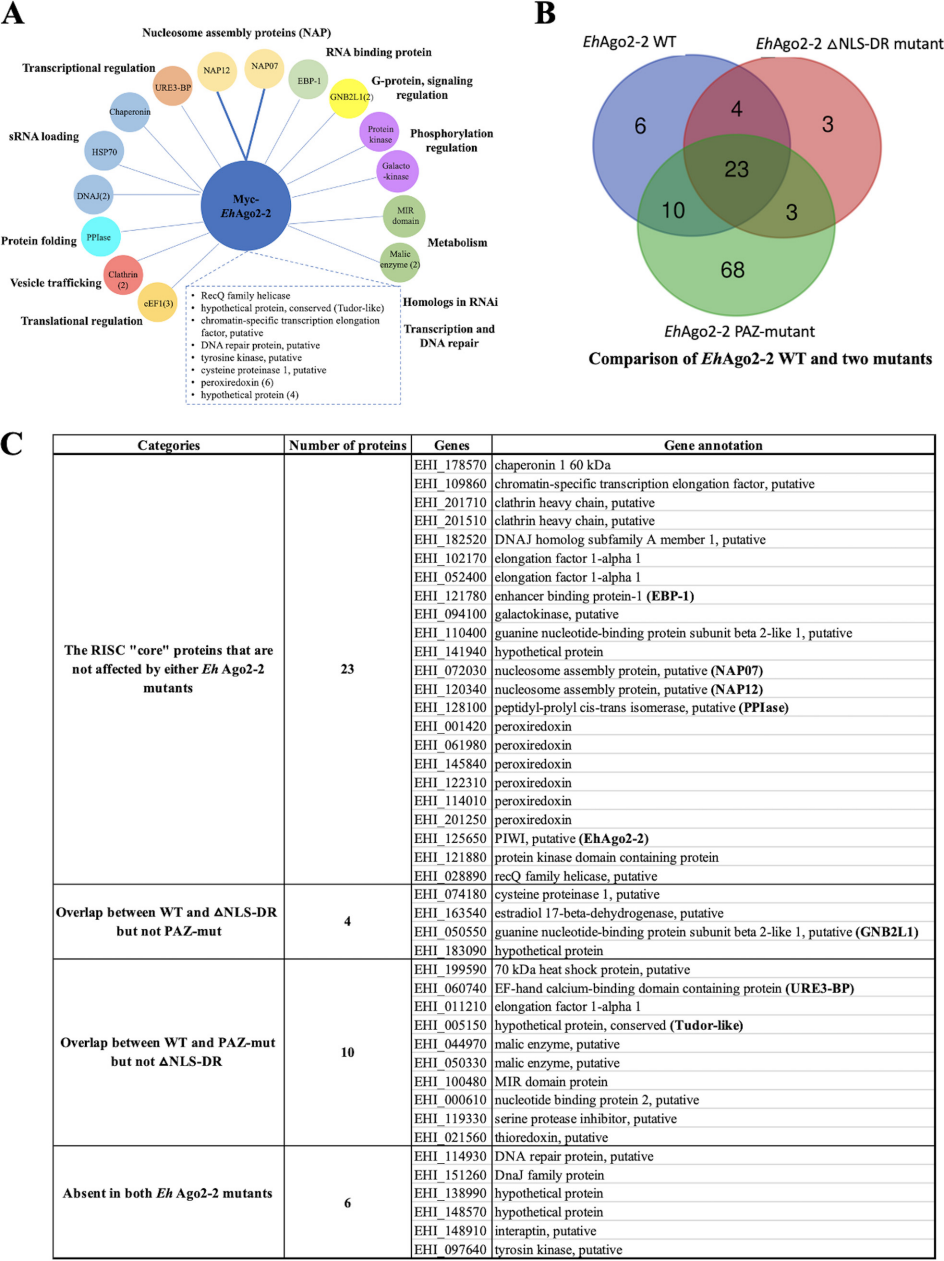
*Eh*Ago2-2 RISC components and their potential cellular function(s). (A) Diagram of the 43 protein hits from Myc-*Eh*Ago2-2 RISC. High/moderate-confidence hits are represented by colored bubbles and grouped by functions; low-confidence hits are listed in the dashed-line box. Protein functions are based on either current gene annotation in the genome or InterPro protein domain indicated in AmoebaDB. Protein hits identified in this study exhibit a broad range of biological activities, including transcriptional and translational regulation, sRNA loading, and cell signaling. There are two proteins with NAP domains (shown as thick lines) which, interestingly, have not previously been observed in RISCs from other studied systems. RecQ helicase and Tudor-like protein are the only two protein hits that can be linked to known RNAi factors found in model systems. Both the RecQ helicase and Tudor-like protein were identified as low-confidence hits, as shown in the dashed-line box. (B and C) Three-way comparison of RISC components among *Eh*Ago2-2 WT and its two mutants. Mass spectrometry analyses pinpointed the RISC components affected in these two functional mutants. There are 23 protein hits found in all three samples, suggesting that they are likely core components of Ago RISC. Each mutant also shows a small set of proteins whose association with RISC was affected: four protein hits are PAZ-mut specific (overlap between WT and ΔNLS-DR mutant); 10 protein hits (notably URE3-BP and Tudor-like protein) are ΔNLS-DR mutant specific (overlap between WT and PAZ-mut); six protein hits are absent in both mutants.

To better understand the 43 *Eh*Ago2-2 RISC components identified, we investigated their potential cellular function(s) based on current genome annotations or functional domains (InterPro domain in AmoebaDB). Figure 2A diagrams the functional annotations of these RISC components using a colored bubble plot. The protein hits are annotated with a diverse array of functions, including transcriptional and translational regulation, sRNA loading, RNA binding, vesicle trafficking, and protein folding and modification, and may be involved in cell signaling and metabolic pathways. Interestingly, several conserved RISC components associated with mammalian systems, such as Dicer, transactivating response RNA-binding protein (TRBP), GW182/TNRC6B, or exportin 8, were not identified in our study. This is in agreement with our *in silico* bioinformatic search of the *Entamoeba* genome; these mammalian RISC components are conspicuously absent from the *Entamoeba* genome (29). However, considering that the E. histolytica genome is not well characterized, with more than half of the predicted genes being hypothetical (53%), we cannot definitively rule out the absence of canonical RISC machinery found in model organisms. The other RNAi machinery components encoded by the E. histolytica genome are *Eh*RdRPs (EHI_139420, EHI_179800, and EHI_086260) and *Eh*Agos (*Eh*Ago2-1, -2-2, and -2-3) (29). However, none of these known RNAi proteins were identified in our *Eh*Ago2-2 RISC data set, possibly due to low expression of these proteins (*Eh*RdRPs and two other *Eh*Agos) in the parasite (29). Alternatively, *Eh*RdRPs or *Eh*Agos could form separate functional complexes outside the *Eh*Ago2-2 RISC.

Tudor-like protein (EHI_005150, a hypothetical protein with a Tudor domain by E value 2.1e−10) and RecQ helicase (EHI_028890) were identified in our analysis and could be potential homologs of known RISC components in model systems. The Tudor domain protein is a highly conserved Ago/Piwi RISC component found in C. elegans, *Drosophila*, and mammals (37). QDE-3, one of the RecQ family proteins, is an important RNAi factor involved in quelling in *Neurospora*. The rat rRecQ-1, a homolog of QDE-3, is found to be associated with Piwi RISC (18). The presence of two highly conserved RISC components in our data set validates our approach toward revealing the true interactome of RISC components in E. histolytica. Further studies are needed to elucidate the exact functions of E. histolytica Tudor-like protein and RecQ helicase and their roles in the RNAi pathway.

*Eh*Ago2-2 RISC contains a set of chaperone proteins (Hsp70, DnaJ homolog subfamily A member 1, and chaperonin 1) reminiscent of RISC chaperone machinery proteins (Hsc70/Hsp90 and others) found in model systems (38). RISC formation often involves multiple steps, such as loading of sRNA duplexes, unwinding, and passenger strand cleavage (39). Identification of chaperone machinery proteins in *Eh*Ago2-2 RISC indicates that a similar process of sRNA loading may exist in the parasite. However, Hsp90 is not seen in our data set despite being highly expressed in E. histolytica (40). During heat stress, E. histolytica parasites upregulate many chaperone proteins, including Hsp90 and multiple Hsp70s, as well as cofactors Hsp40 (DnaJ), BAG-1, and CHIP (41). The *Eh*Ago2-2 RISC chaperones found in this study are different from those found during heat stress, suggesting that the two different biological events/processes involve different sets of chaperone proteins.

*Eh*Ago2-2 RISC contains two NAP domain proteins: EHI_072030 and EHI_120340 (which we will refer to as NAP07 and NAP12, respectively). It is worth noting that these two proteins were among the top-ranked protein hits in all three replicates of *Eh*Ago2-2. NAP domain proteins, functioning in nucleosome formation or as histone chaperones, have not been previously identified in other RNAi systems (42). Searching the E. histolytica’s genome revealed 11 potential proteins with annotated NAP domains. Phylogenetic comparison and alignment analysis using Clustal Omega showed the divergence of *Eh*NAPs from NAPs found in model systems (see Fig. S3A). Compared with yeast NAP1 (yNAP1), all *Eh*NAPs are relatively short, lacking ∼60 residues at the N terminus (similar to Caenorhabditis elegans NAP1 [*Ce*NAP1]) (Fig. S3B). Some *Eh*NAPs, including NAP07 and NAP12, appear to also lack the acidic C-terminal tail (Fig. S3B and C). The acidic C-terminal tail is a unique characteristic of NAP proteins which contributes to histone binding (43). Future studies will be required to examine the function of NAP07 and NAP12 in *Entamoeba*, specifically their activity pertaining to RNAi. These studies could also elucidate NAP07 and NAP12’s potential involvement in histone binding or nucleosome formation.

10.1128/mBio.01540-21.3 FIG S3 Phylogenetic analysis of E. histolytica NAPs and sequence alignment analysis. The genome of E. histolytica HM-1:IMSS contains 11 potential proteins that have an annotated NAP domain (https://amoebadb.org). The phylogenic tree is derived by sequence alignment analysis using Clustal Omega. (A) The divergence of *Eh*NAPs from model systems (yeast NAP1, human NAP1, and C. elegans NAP1). NAP07 and NAP12 (boxed) are divergent from NAP1 from model systems (solid triangles). (B and C) Sequence alignment analysis by Clustal Omega shows that NAP07 and NAP12 lack an N-terminal sequence compared with yeast NAP1 (first 60 aa, boxed in blue). Another noticeable feature is in the C-terminal tail, where NAP07 and NAP12 lack an acidic C-terminal tail (boxed in red). The acidic residues are thought to contribute to histone binding function in model systems. Download FIG S3, PDF file, 0.6 MB. Copyright © 2021 Zhang et al. 2021 Zhang et al. https://creativecommons.org/licenses/by/4.0/ This content is distributed under the terms of the Creative Commons Attribution 4.0 International license.

Lastly, our Ago RISC analysis indicated that the known transcription factor URE3-BP (upstream regulatory element 3-binding protein) (EHI_060740) is a major *Eh*Ago2-2 RISC component. URE3-BP was previously identified as a calcium-responsive regulator for two virulence genes: *Hgl5* (Gal/GalNAc lectin) and *Fdx1* (ferredoxin) (44). URE3-BP broadly regulates parasite virulence, as demonstrated by transcriptome profiling both *in vitro* and *in vivo* (45, 46). Thus, the amebic RNAi pathway likely participates in the regulation of genes that control parasite virulence.

### RISC components of two Ago mutants.

Our study also included two functional *Eh*Ago2-2 mutants to pinpoint the effects of these mutants on the RISC contents. These two mutant proteins have either impaired nuclear localization (Myc-*Eh*Ago2-2^ΔNLS-DR^) or impaired sRNA binding (Myc-DHFR-*Eh*Ago2-2^PAZ-mut^) (30). Using a similar strategy of IP mass spectrometry analysis, we found 32 exclusive protein hits and one enriched hit for *Eh*Ago2-2^ΔNLS-DR^ RISC (Fig. S2B and Table S1A) and 98 exclusive hits and 6 enriched hits for the *Eh*Ago2-2^PAZ-mut^ RISC (Fig. S2C and Table S1B).

We performed three-way comparisons of the RISC data sets (shown in Fig. 2B and C) and identified 23 protein hits shared among all three data sets. These 23 proteins that were unaffected by either mutant are likely bona fide components of Ago RISC. Furthermore, these 23 proteins were cross-confirmed by nine mass spectrometry data sets in our study. Among these hits, the two NAPs (NAP07 and NAP12) remained as high-confidence hits in all three samples, indicating a strong association to Ago. There were four proteins affected by the PAZ mutant (overlap between the WT and the ΔNLS-DR mutant but not the PAZ-mut mutant) and 10 proteins affected by the ΔNLS-DR mutant (overlap between WT and PAZ-mut but not ΔNLS-DR), as listed in the Fig. 2C. Finally, six proteins were absent in both mutants, including DnaJ family protein and DNA repair protein (EHI_114930).

We noticed several protein hits against WT Ago RISC were ranked differently under the two mutant conditions, which could be interpreted as association-level changes within RISC. We observed six high-confidence protein hits in the WT (two malic enzymes, two DnaJ proteins, MIR domain, and URE3-BP) were absent or became low-confidence hits in the two mutants. The absence of URE3-BP in both mutants is interesting, as this transcription factor is a key regulator for amebic virulence. The *Eh*Ago2-2^ΔNLS-DR^ mutant had a substantial effect on the Ago RISC composition: redox proteins were altered (all six low-confidence ranked peroxiredoxin hits became high/medium hits in the mutant), and several high-confidence hits were lost, including enhancer binding protein-1 (EBP-1), Hsp70, and elongation factor 1-alpha 1. For the *Eh*Ago2-2^PAZ-mut^ mutant, we noticed that it contained many hits associated with proteins involved in the ubiquitination pathway (Table S1B). This observation could be due to the overexpressed mutant protein triggering a protein degradation pathway in the parasite.

### Cross-validation of Ago RISC components by tagging additional RNAi pathway protein.

**FIG 3:**
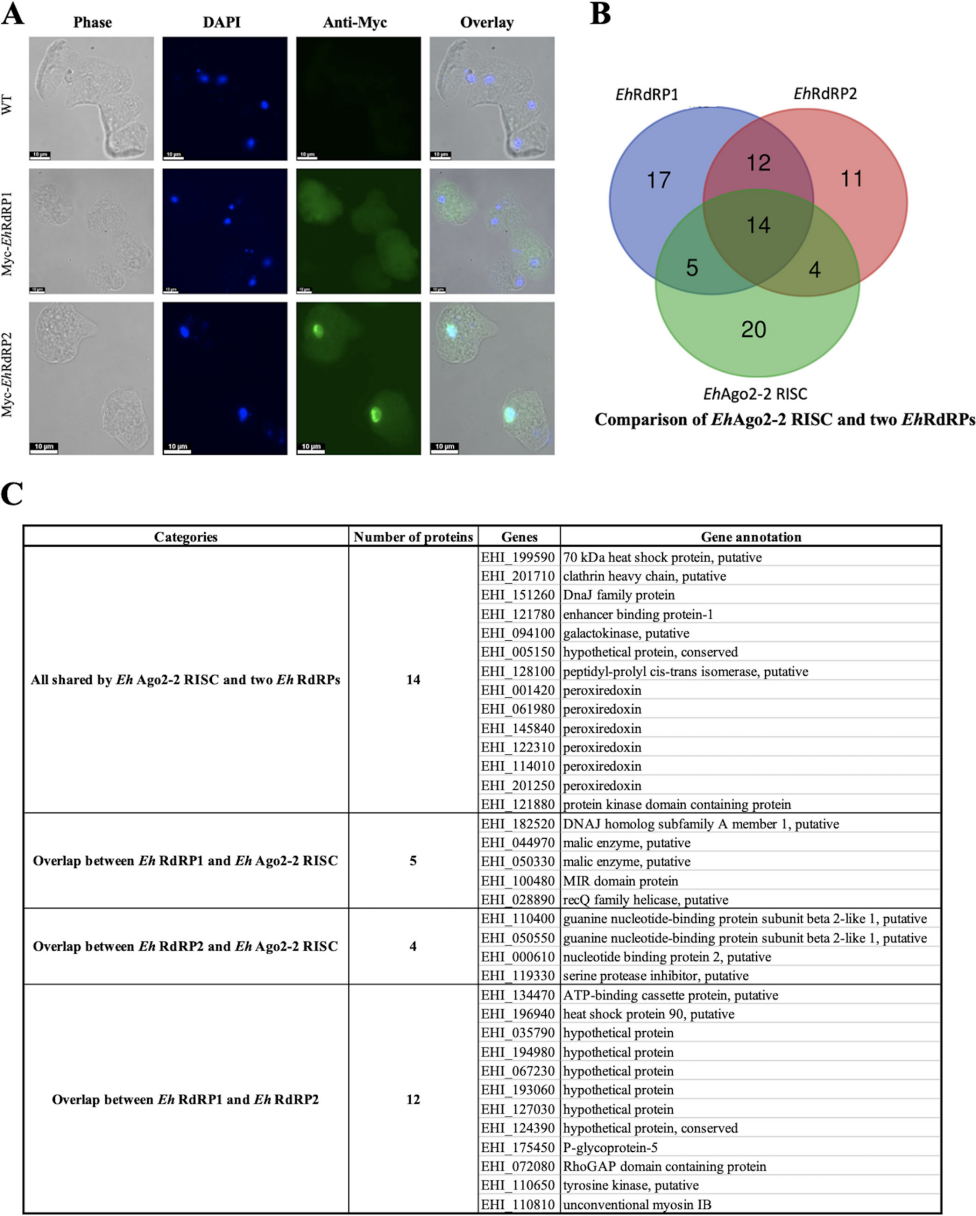
Characterization of additional RNAi pathway proteins, *Eh*RdRPs, for protein localization and IP mass spectrometry analyses. (A) Protein localization of two *Eh*RdRPs. Parasite cells were fixed using methanol-acetone (1:1) and incubated with mouse anti-Myc antibody and subsequently with goat anti-mouse antibody conjugated to Alexa 488. IFAs show that overexpressed Myc-tagged *Eh*RdRP1 distributes ubiquitously in the cell, while Myc-tagged *Eh*RdRP2 is enriched in the nucleus with faint cytoplasmic staining, a pattern similar to that in Myc-tagged *Eh*Ago2-2 cells (31). (B and C) IP mass spectrometry analyses of *Eh*RdRPs for their interacting partners which overlap mostly with Ago RISC. Using the same selection criteria established for Ago RISC, we performed three replicates of IP mass spectrometry analyses for each *Eh*RdRP. There were 48 and 41 exclusive hits for *Eh*RdRP1 and *Eh*RdRP2, respectively. Both *Eh*RdRP data sets overlap that for *Eh*Ago2-2 RISC (with more than 50% protein hits, as shown in panel B), indicating RNAi function in E. histolytica may involve these RNAi protein complexes to function together. The shared components are listed in a table in panel C, showing many aforementioned functional groups of *Eh*Ago2-2 RISC, such as RNA binding proteins, chaperones for sRNA loading, and RNAi homologs (RecQ helicase and Tudor-like protein).

To validate the identified *Eh*Ago2-2 RISC components, we examined other known RNAi pathway proteins in E. histolytica. Among RNAi pathway proteins found in this parasite are Agos and RdRPs. The RdRP proteins function in the RNAi gene silencing pathway and are responsible for the biogenesis of secondary sRNAs in yeast, C. elegans, and plants (35). It has been shown that RdRPs are tightly linked to Ago RISC in yeast and C. elegans by mass spectrometry analysis (15, 47). Thus, we selected two *Eh*RdRPs (EHI_139420 and EHI_179800) and overexpressed Myc-tagged *Eh*RdRP proteins and confirmed their expression by Western blotting (see Fig. S4A). Immunofluorescence assay (IFA) analysis (Fig. 3A) of Myc-*Eh*RdRP1 shows whole-cell staining, while Myc-*Eh*RdRP2 shows nuclear staining with a faint signal in the cytoplasm, a pattern similar to that in *Eh*Ago2-2 cells (31). We performed IP and mass spectrometry analysis for the Myc-tagged *Eh*RdRPs (Fig. S4A) and found 48 exclusive protein hits in the *Eh*RdRP1 sample and 41 exclusive protein hits in the *Eh*RdRP2 sample (Fig. S4B and C; Table S2). We noticed that the two *Eh*RdRP data sets overlap extensively. Both *Eh*RdRP data sets also overlap the *Eh*Ago2-2 RISC data set, with greater than 50% protein hits (23/43), as shown in Fig. 3B. This suggests that these protein complexes could function together to carry out RNAi in E. histolytica. These shared components are listed in Fig. 3C, including RNA binding proteins, chaperones for sRNA loading, and RNAi homologs (RecQ helicase and Tudor-like protein). It is worth noting that *Eh*RdRP1 possibly interacts with another Ago protein (*Eh*Ago2-3), indicating a broader RNAi network among all three Ago proteins in the parasite. Of note, we did not identify *Eh*Ago2-1 in these IP samples, but it is worth noting that this protein exhibits very low expression, which may have limited its identification by IP and mass spectrometry.

10.1128/mBio.01540-21.4 FIG S4 Characterization of additional RNAi pathway proteins, *Eh*RdRPs, by anti-Myc IPs and mass spectrometry analyses. (A) Western blots and silver staining for the anti-Myc IP samples of two *Eh*RdRPs. Similar to the *Eh*Ago2-2 anti-Myc IP shown in Fig. S1, the anti-Myc Western (top) shows expression of Myc-*Eh*RdRPs at their expected sizes (134 kDa for Myc-*Eh*RdRP1 and 116 kDa for Myc-*Eh*RdRP2). Specific pulldown of Myc-tagged proteins is seen in the IP elution lanes but not in the control IP. Anti-actin Western blotting shows that the actin signal is almost depleted in IP elution lanes compared with that in lanes for input lysate and IP supernatant. (Bottom) Silver staining monitoring IP elution samples of *Eh*RdRPs. The thick band at 50 kDa is IgG from the beads. Arrows indicate the enriched protein bands for *Eh*RdRPs in IP elution samples. (B and C) Identification of protein hits by Venn diagram analysis for two *Eh*RdRPs. Based on the same criteria used for *Eh*Ago2-2 data set analysis, we analyzed the three replicates of each *Eh*RdRP and identified 48 exclusive protein hits for *Eh*RdRP1 and 41 exclusive protein hits for *Eh*RdRP2. Download FIG S4, PDF file, 2.5 MB. Copyright © 2021 Zhang et al. 2021 Zhang et al. https://creativecommons.org/licenses/by/4.0/ This content is distributed under the terms of the Creative Commons Attribution 4.0 International license.

10.1128/mBio.01540-21.9 TABLE S2 List of protein hits for two *Eh*RdRPs that were identified by mass spectrometry analysis. (A) List of *Eh*RdRP1 protein hits identified by mass spectrometry analysis. (B) List of *Eh*RdRP2 protein hits identified by mass spectrometry analysis. Download Table S2, XLSX file, 0.01 MB. Copyright © 2021 Zhang et al. 2021 Zhang et al. https://creativecommons.org/licenses/by/4.0/ This content is distributed under the terms of the Creative Commons Attribution 4.0 International license.

To reveal the protein-protein interactions of the Ago RISC components, we chose five protein hits found within this study for further characterization. NAP07 and NAP12 appear to be novel and unique to the parasite RISC. EBP-1 contains an RNA-binding motif, which could be important for sRNA or RISC-targeted RNAs. Guanine nucleotide-binding protein subunit beta 2-like 1 (GNB2L1), also known as receptor for activated C kinase 1 (RACK1), belongs to the tryptophan-aspartate repeat (WD-repeat) family proteins and functions as a master regulator for multiple cellular processes in other systems (48). Lastly, Tudor-like domain protein was also selected, as it is likely a homolog component of Ago RISC in model systems.

**FIG 4:**
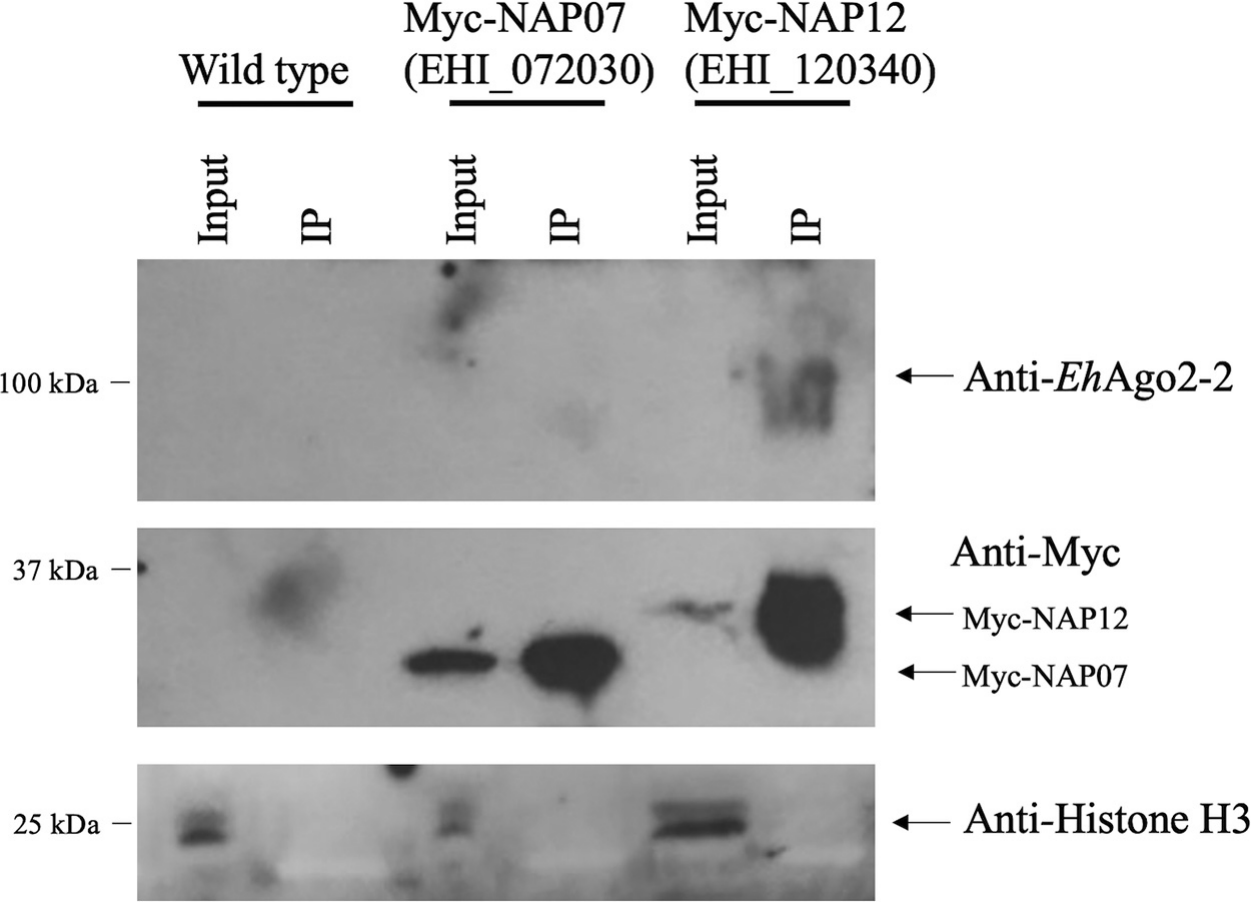
Western blotting of co-IP samples detected the interaction of NAP12 with *Eh*Ago2-2. We characterized five additional protein hits from our initial Ago RISC analyses. We performed anti-Myc co-IP experiments for each of the five Myc-tagged protein cell lines. *Eh*Ago2-2 was detected in the co-IP for Myc-NAP12 using a custom-generated anti-*Eh*Ago2-2 antibody. *Eh*Ago2-2 signal can be detected in the anti-Myc IP sample for Myc-NAP12 but not in the IP samples for Myc-NAP07 or for the other three Myc-tagged proteins (see also Fig. S5 in the supplemental material). Due to low protein loading, a faint *Eh*Ago2-2 signal was detected only upon overnight exposure for input lanes (data not shown). Anti-Myc Western blotting detected signals for Myc-tagged NAP07 and NAP12 proteins at their expected sizes. The membrane was also probed with an anti-histone 3 antibody, which showed signal in all input lanes but not in the IP lanes.

Similar to the approaches used before, the five selected genes were cloned into the same plasmid vector for Myc-tagged overexpression. After transfecting the constructs into parasites and confirming the expression of each Myc-tagged protein, we then tested if endogenous *Eh*Ago2-2 can be pulled down in a co-IP experiment using a ready-made custom antibody against *Eh*Ago2-2 (the antibody was generated and validated in our previous study [31]). Figure 4 and Fig. S5 show that *Eh*Ago2-2 was only detected in the Myc-NAP12 co-IP sample (Fig. 4) and not in the co-IP samples of the other four proteins (Fig. S5). This indicated that a direct association of NAP12 to *Eh*Ago2-2 can be verified under the co-IP experiment conditions.

10.1128/mBio.01540-21.5 FIG S5 *Eh*Ago2-2 is not detected in the co-IPs of three cell lines of Myc-EBP-1, Myc-GNB2L1, and Myc-Tudor-like protein. Similarly to those in Fig. 4, co-IP samples for these three Myc-tagged protein cell lines were probed for *Eh*Ago2-2 presence using a custom-generated *Eh*Ago2-2 antibody. *Eh*Ago2-2 signal is absent in all three anti-Myc IP samples. Anti-Myc Westerns blots show the specific enriched signal for each tagged protein, while anti-actin Westerns blots show the depletion of this abundant protein signal in IPs for those samples. Download FIG S5, PDF file, 0.2 MB. Copyright © 2021 Zhang et al. 2021 Zhang et al. https://creativecommons.org/licenses/by/4.0/ This content is distributed under the terms of the Creative Commons Attribution 4.0 International license.

As mass spectrometry analysis can provide a sensitive means to broadly identify potential interacting proteins from an IP sample, we performed two independent IPs for each of the five Myc-tagged proteins to identify the interacting network among these RISC components. We set the initial selection criteria to require two of both replicates for each sample and focused on exclusive hits after comparison with the Luc control. We then compared the exclusive hits from each tagged protein data set to the *Eh*Ago2-2 RISC hits. For example, the Myc-NAP07 mass spectrometry analysis (shown in Fig. S6A) generated 75 exclusive protein hits after removal of the Luc control. There were 19 protein hits that overlapped between Myc-NAP07 and *Eh*Ago2-2 RISC data sets, as listed in the inset table of Fig. S6A (with the high-confidence hits as green). The analyses for NAP12, EBP-1, GNB2L1, and Tudor-like domain protein IP samples are shown in Fig. S6B to E.

10.1128/mBio.01540-21.6 FIG S6 Mass spectrometry analyses for IP samples of five Myc-tagged protein cell lines (Myc-NAP07, Myc-NAP12, Myc-EBP-1, Myc-GNB2L1, and Myc-Tudor-like protein). Venn diagrams depict data processing workflow: for each cell line, two IP replicates were performed for mass spectrometry analyses. The nonribosomal hits present in two of two replicates were selected for further analysis. Exclusive hits were obtained by comparison with the Luc control. Exclusive hits for each cell line were compared to the 43 *Eh*Ago2-2 RISC hits, and overlapping hits were analyzed, suggesting potential interactions with Ago RISC. (A) Myc-NAP07 IP mass spectrometry analyses show 11 Ago RISC components (listed in the inset table as high-confidence hits in green). (B to E) The same data analysis flow was used for Myc-NAP12, Myc-EBP-1, Myc-GNB2L1, and Myc-Tudor-like data analyses, respectively. Download FIG S6, PDF file, 1.1 MB. Copyright © 2021 Zhang et al. 2021 Zhang et al. https://creativecommons.org/licenses/by/4.0/ This content is distributed under the terms of the Creative Commons Attribution 4.0 International license.

**FIG 5:**
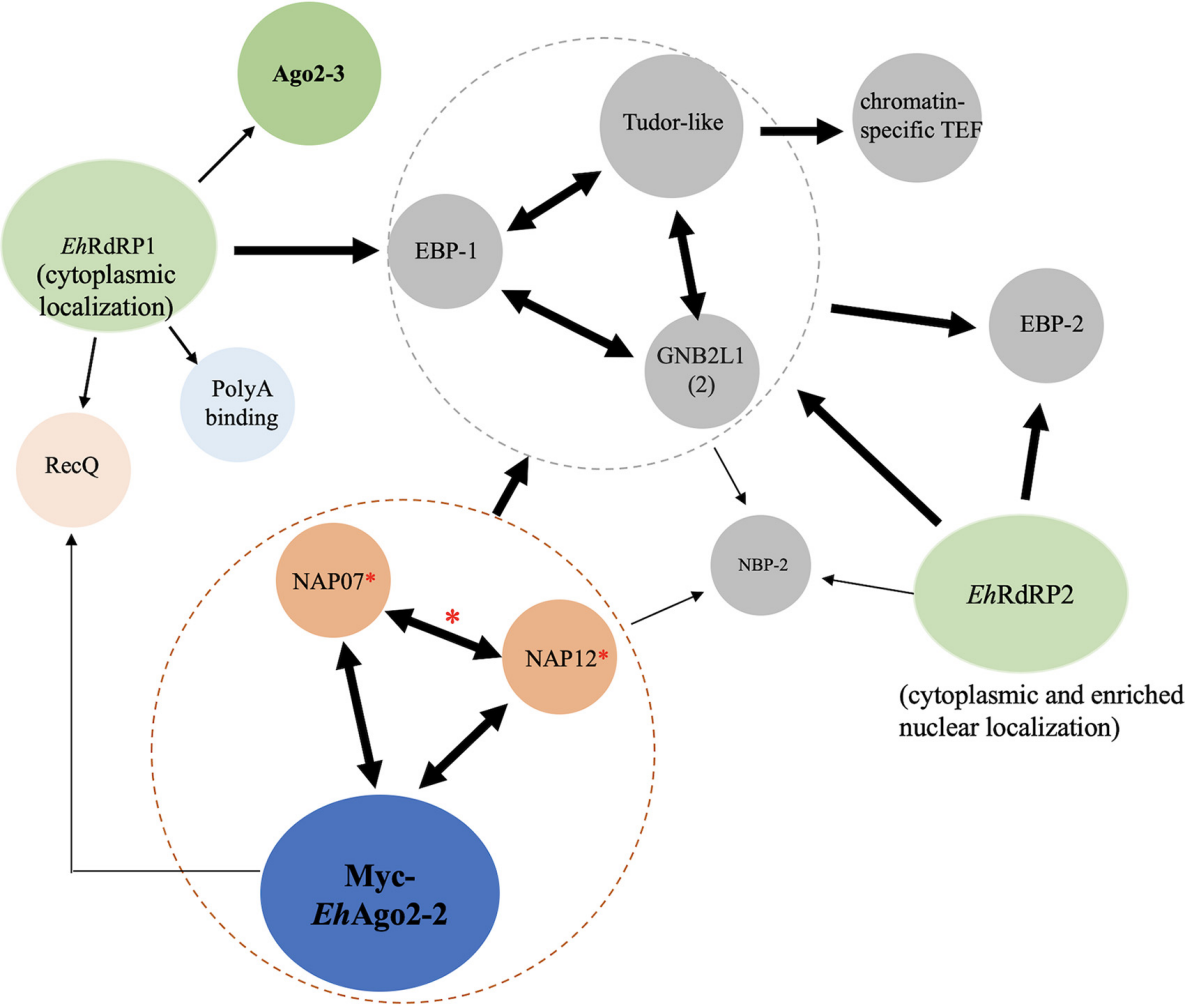
Major interactome revealed by mass spectrometry analyses of *Eh*Ago2-2, *Eh*RdRPs, and five selected Ago RISC proteins. In addition to previous *Eh*Ago2-2 and *Eh*RdRP cell lines, mass spectrometry analyses were performed for each cell line overexpressing five putative Ago RISC components. Mass spectrometry analysis for each cell line was performed in duplicates. The detailed Venn diagram analysis is shown in Fig. S5. The major protein-protein interactions can be summarized as follows: *Eh*Ago2-2, NAP07, and NAP12 form a closely interacting group (in brown circle) with reciprocal pulldowns among three proteins, while a second interacting group (in gray circle) consists of EBP-1, Tudor-like protein, and GNB2L1, with reciprocal pulldowns among these three proteins. The two *Eh*RdRP complexes interact with *Eh*Ago2-2 RISC component proteins of EBP-1, Tudor-like protein, and GNB2L1. *Eh*RdRP1 and *Eh*Ago2-2 both interact with RecQ helicase, a RISC homolog identified in this study. Other interesting RNA binding protein hits are *Eh*Ago2-3 and poly(A) binding proteins in the *Eh*RdRP1 data set and EBP-2 and NBP-2 in the *Eh*RdRP2 data set. Single arrow indicates one-way pulldown; double arrow indicates reciprocal pulldown. Dashed circles indicate proteins are grouped as closely interacted proteins. Thick arrow lines indicate high-confidence hits, thin arrow lines indicate low-confidence hits, and * indicates a recombinant protein was generated in a bacterial system and the protein interaction was confirmed (see Fig. 6).

To summarize the overall findings of the mass spectrometry analyses of these five Myc-tagged proteins, we listed the major protein-protein interacting relationships with *Eh*Ago2-2 RISC in Fig. 5. (i) Each of the five selected proteins had many proteomic hits (including some found in the *Eh*Ago2-2 RISC), indicating that each of these five proteins could have broad cellular functions in addition to their involvement in RNAi. (ii) Two NAP proteins reciprocally pulled down each other, indicating a strong binding of two NAPs. In addition, both NAPs pulled down *Eh*Ago2-2. (iii) *Eh*Ago2-2 and the two NAP proteins interacted with EBP-1, GNB2L1, and Tudor-like proteins. (iv) The EBP-1, GNB2L1, and Tudor-like proteins exhibited reciprocal pulldown with each other. Thus, we have identified two groups of closely interacting proteins within the Ago RISC (circled in brown and gray in Fig. 5). (v) Two *Eh*RdRPs interacted mainly with three RISC components (EBP-1, GNB2L1, and Tudor-like proteins) (the brown circle in Fig. 5). Overall, our data define the major interactome of Ago RISC components and establish a protein-protein interaction map for these RISC components in relation to Ago.

### *In vitro* assays confirm a direct interaction between two NAP proteins.

**FIG 6:**
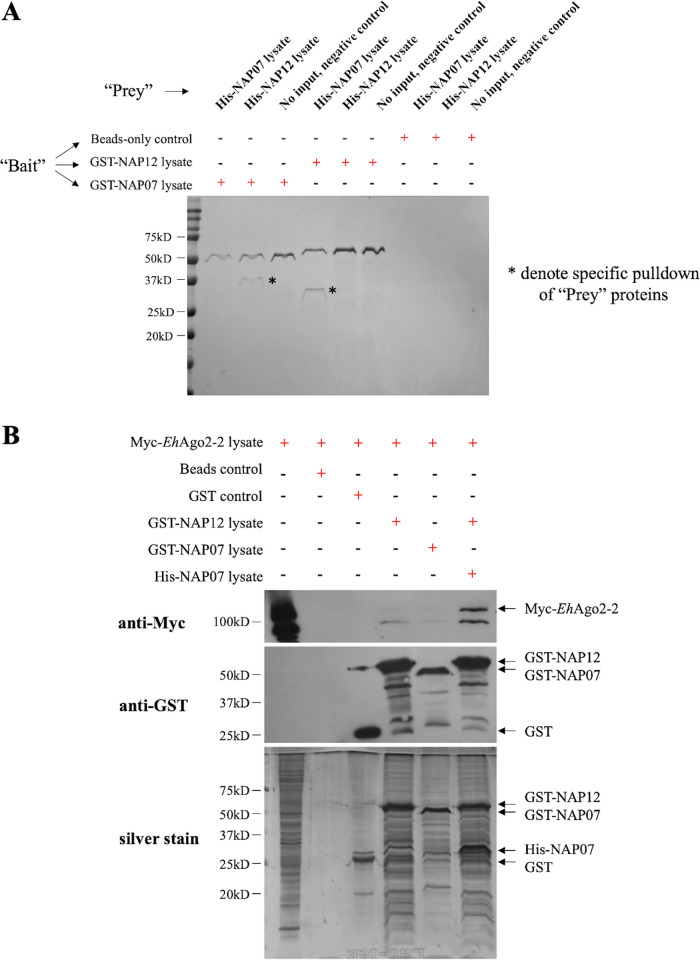
*In vitro* pulldown assay confirms a direct interaction between two NAP proteins and their interaction with *Eh*Ago2-2. (A) A physical interaction exists between recombinant NAP proteins by GST pulldown. The GST pulldown assay was performed using glutathione beads to bind “bait” GST-tagged NAP proteins from the bacterial lysate. The beads were washed and then incubated with bacterial lysates containing “prey” His-tag NAP proteins. After washes, beads were then eluted by GST elution buffer, and the sample was collected. Reciprocal pulldowns of His-NAP12 (expected size, 35 kDa) using “bait” GST-NAP07 (expected size, 54 kDa) and pulldown of His-NAP07 (expected size, 32 kDa) using “bait” GST-NAP12 (expected size, 57 kDa) were observed, the pulldown of each “prey” protein is indicated by an asterisk. The beads-only and negative input controls worked as expected, as there is no pulldown of any “prey” proteins. The control GST-tag alone does not pulldown either of the His-tagged NAP proteins; using His-tagged ERM-BP (an irrelevant amebic protein [49]), all “bait” proteins of GST-tagged NAPs showed no reactivity. All controls are shown in Fig. S7C. (B) A physical interaction exists between *Eh*Ago2-2 and recombinant NAP proteins by *in vitro* pulldown of amebic cell lysate. GST-NAP07 and GST-NAP12 were first immobilized onto glutathione-agarose beads as “bait” proteins. Beads preloaded with “bait” proteins were incubated with cell lysates prepared from parasites overexpressing Myc-*Eh*Ago2-2. After incubation and washing, the beads were eluted and samples were analyzed. Western blotting using mouse anti-Myc and mouse anti-GST antibodies was also performed. There is a noticeable Myc-*Eh*Ago2-2 signal in samples containing NAP “baits” (the rightmost three lanes). Two bands are usually observed due to a degraded protein form of Myc-Ago2-2. No signal corresponding to Myc-*Eh*Ago2-2 was observed in the beads-only or GST controls. Arrows are pointing to the detected proteins under each condition.

To validate a direct physical interaction between the two NAP proteins, we expressed the two NAPs in a bacterial system, with both N-terminal glutathione-S-transferase (GST) tags and His tags. All four recombinant proteins showed prominent bands by SDS-PAGE at their expected sizes upon induction with isopropyl-β-d-thiogalactopyranoside (IPTG) (see Fig. S7A). Using the solubilization and purification conditions outlined in Materials and Methods, we were able to purify both GST-tagged and His-tagged NAP proteins (Fig. S7B). To test if there is a specific interaction between two NAP proteins, we performed a GST pulldown assay. GST-tagged NAPs were used as “bait” proteins and incubated with bacterial lysates containing “prey” His-tagged NAPs. Figure 6A shows a specific pulldown of His-NAP12 using bait GST-NAP07 and pulldown of His-NAP07 using bait GST-NAP12. The controls showed no interaction with any of these NAP proteins (additional controls are negative, as shown in Fig. S7C, including a pulldown assay using bait GST tag or pulldown for irrelevant His-tagged ERM-BP protein [49] lysate). Thus, a direct interaction between NAP07 and NAP12 can be confirmed using bacterial expression systems.

10.1128/mBio.01540-21.7 FIG S7 Bacterial expression of recombinant proteins of two NAP proteins. (A) GST-tagged proteins (GST-NAP07 and GST-NAP12) and His-tagged proteins (His-NAP07 and His-NAP12) were expressed upon induction with IPTG. Cells before and after induction were lysed using 1× Laemmli buffer, and total protein was separated by SDS-PAGE. The arrows indicate the expected protein sizes: GST-NAP07 at 54 kDa; GST-NAP12 at 57 kDa; His-NAP07 at 32 kDa; His-NAP12 at 35 kDa. (B) Both GST-tagged and His-tagged NAP proteins were solubilized and purified. Using the solubilization and purification conditions (see Materials and Methods), we purified all four NAPS expressed recombinantly in a bacterial system. Samples shown are input, flow through, 1st wash, and elution from each protein purification preparation. SDS-PAGE gel was stained by Coomassie blue G-250. (C) Additional controls for the *in vitro* pulldown assay. The two additional controls were GST-tag alone as “bait” protein and His-tagged ERM-BP (an irrelevant His-tagged amebic protein [49] as “prey” control). As shown on the left, the control “bait” GST tag alone (size, 25 kDa) shows no interaction with either “prey” His-tagged NAP07 or NAP12. On the right, neither “bait” sample (GST, GST-NAP07, or GST-NAP12) showed interaction with the “prey” of the irrelevant amebic protein, His-ERM-BP. Download FIG S7, PDF file, 1.1 MB. Copyright © 2021 Zhang et al. 2021 Zhang et al. https://creativecommons.org/licenses/by/4.0/ This content is distributed under the terms of the Creative Commons Attribution 4.0 International license.

We further set out to test if GST-tagged NAPs can be used as bait proteins to pull down *Eh*Ago2-2 from amebic cell lysates. In this experiment, GST-tagged NAP07 or NAP12 was immobilized onto glutathione-agarose beads as bait proteins. The beads were then incubated with parasite whole-cell lysate prepared from a Myc-*Eh*Ago2-2 overexpression cell line. In addition, we included a prebound NAP bait sample (GST-NAP12 incubated with His-NAP07 lysate to form prebound NAPs of GST-NAP12 with His-NAP07). We analyzed the eluted proteins from the beads by silver staining, which showed prominent bait proteins for each sample at their expected sizes (Fig. 6B, bottom). To check if the prey Myc-*Eh*Ago2-2 was present, we probed the eluted samples by Western blotting using an anti-Myc antibody. We observed Myc-*Eh*Ago2-2 signal in the bait sample lanes of NAP12, NAP07, and prebound NAPs; Myc*-Eh*Ago2-2 was not observed in the control samples. Thus, we have confirmed the interaction between *Eh*Ago2-2 and the two NAPs using a recombinant protein approach.

## DISCUSSION

In this paper, we characterized E. histolytica Ago RISC by IP mass spectrometry and identified 43 protein hits as components of *Eh*Ago2-2 RISC. These 43 protein components have a broad range of functions based on *in silico* analyses and gene annotations. Analyses of two functional *Eh*Ago2-2 mutants (with alterations in small RNA binding and nuclear localization) demonstrated that Ago RISC contents are affected under each mutant condition. Our main findings from this study can be summarized as follows: (i) *Eh*Ago2-2 RISC lacks several known RNAi factors found in typical siRNA/miRNA pathways but contains RecQ helicase and Tudor-like protein, which are reminiscent of Piwi RISC; (ii) *Eh*Ago2-2 RISC contains two unique proteins with NAP domains, and their physical interaction with Ago was confirmed using an *in vitro* recombinant system; (iii) *Eh*Ago2-2 RISC contains a major parasite virulence transcriptional factor; (iv) analyses of *Eh*RdRPs, additional RNAi pathway proteins, identified significant overlap with *Eh*Ago2-2 RISC content; and (v) characterization of five selected proteins from *Eh*Ago2-2 RISC identified protein-protein interacting groups within the Ago RISC. Our work is the first to elucidate Ago RISC components in the protozoan parasite *Entamoeba*. We also defined the interactome of these Ago RISC components and built a map of protein-protein interactions for selected RISC components in relation to Ago.

Ago and its RISC components often display great versatility depending on the Ago type, silencing mechanism, or specific RNAi pathways (50, 51). Among all Ago RISCs studied so far, T. gondii Ago is the only parasitic Ago protein that has been subjected to RISC analysis. Much similarity was drawn between *Tg*Ago and human Ago2 RISC contents, possibly due to the presence of an active miRNA pathway in both organisms (23). Among the 43 protein hits identified as *Eh*Ago2-2 RISC components, most do not share homology to known RNAi factors found in other/model systems. E. histolytica appears to lack a canonical Dicer protein; the only RNase III domain protein (EHI_068740) was previously shown to result in partial cleavage of dsRNA under *in vitro* conditions. However, it was not present in our *Eh*Ago2-2 RISC data set. It remains to be seen if this RNase III domain protein is a necessary factor for RNAi in the parasite. Our previous work revealed all three *Eh*Agos bind to sRNA with a 5′-polyphosphate structure, indicating these sRNAs could be generated by RdRP in this parasite (28). In this study, we included IP mass spectrometry analysis for two *Eh*RdRPs and found many shared protein hits that also overlap *Eh*Ago2-2 RISC content. It is possible that Ago and RdRP protein complexes orchestrate a coordinated way to effectively carry out RNAi in E. histolytica.

Eukaryotic Ago proteins are grouped into three clades: Ago-like, Piwi-like, and WAGO subfamilies. The three *Eh*Ago proteins are related to the PIWI-like clade by sequence alignment analysis (2, 52). Several studies of Piwi RISC showed no interaction with Dicer or TRBP. However, Piwi RISC interacted with cofactors such as HP1 (heterochromatic protein 1a in flies) (19), rRecQ1 (a DNA nuclease in rats) (18), PRMT5 (an arginine methyltransferase in mice), and a Tudor domain protein (14). The two potential homologs, RecQ family helicase and Tudor-like protein, were identified in *Eh*Ago2-2 RISC, suggesting a similar functional role may also occur and be required for RNAi in this parasite. Future studies geared toward understanding the exact function of these two homologs in E. histolytica could be an exciting avenue to pursue.

We have provided the first account of studying Ago RISC in *Entamoeba* parasites. Our data revealed 43 protein hits with functions covering transcriptional and translational regulation as well as sRNA loading and other cellular functions. This finding suggests multiple cellular processes may be regulated or involved in regulating RNAi in E. histolytica. Future studies of *Eh*Ago RISC components and their interactomes will be crucial to understanding the regulatory mechanisms and biological implications of RNAi in this parasite. Our findings add to the understanding of this basic biological process and expand the current knowledge on RNAi RISC to a deep-branching single-celled eukaryote.

## MATERIALS AND METHODS

### Parasite culture, plasmids and cell lines.

The E. histolytica trophozoites (HM-1:IMSS) were grown axenically under standard conditions as described previously (28, 53). All plasmids used in parasites are based on the pKT3M plasmid, which overexpresses a protein of interest with an N-terminal Myc tag under the control of a cysteine synthase (CS) promoter. The plasmids for overexpressing wild-type *Eh*Ago2-2 and its two mutants are pKT3M-*Eh*Ago2-2, pKT3M*-Eh*Ago2-2^ΔNLS-DR^, and pKT3M-DHFR-*Eh*Ago2-2^PAZ-mut^, respectively. These plasmids were generated in our previous studies (28, 30) and retransfected for use in this study; pKT-CS-Luc overexpresses a full-length luciferase under the same CS promoter but lacks a Myc tag. In addition, we selected two *Eh*RdRP genes (EHI_139420 and EHI_179800) and five genes of RISC components (EHI_072030, EHI_120340, EHI_121780, EHI_050550, and EHI_005150) for further characterization. We cloned each full-length gene into pKT3M, and all plasmid constructs were verified by DNA sequencing. Following transfection, stable cell lines were generated and maintained with 6 μg/ml G418. For bacterial expression of recombinant proteins, two NAP genes (EHI_072030 and EHI_120340) were amplified and cloned in-frame into pGEX-4T-1 (GST tag) using EcoRI and XhoI sites and pET-28b (His tag) using Eco53kI and XhoI sites. The resulting plasmids for N-terminal GST tagging are named pGEX-NAP07 and pGEX-NAP12; plasmids for N-terminal His tags are named pET-NAP07 and pET-NAP12. All primers for cloning used in this study can be found in Table S3 in the supplemental material.

10.1128/mBio.01540-21.10 TABLE S3 List of primers/probes used in this study. Download Table S3, XLSX file, 0.01 MB. Copyright © 2021 Zhang et al. 2021 Zhang et al. https://creativecommons.org/licenses/by/4.0/ This content is distributed under the terms of the Creative Commons Attribution 4.0 International license.

### Parasite cell lysate and IP.

We used a similar protocol as that described in reference 54, with minor modifications. Two T25 flasks of confluent parasite culture were lysed in 3 ml lysis buffer (20 mM Tris-HCl [pH 7.5], 1 mM MgCl_2_, 10% [vol/vol] glycerol, 50 mM NaCl, 0.5% [vol/vol] Nonidet P-40 (NP-40), 1 mM NaF, 1 mM dithiothreitol [DTT], 1 mM phenylmethylsulfonyl fluoride [PMSF], 2× Halt EDTA-free protease inhibitors [Thermo Fisher Scientific] and RNase inhibitor [1 unit/ml]). After incubation on ice for 15 min, the cell lysate was centrifuged at 10,000 rpm for 20 min at 4°C, and the supernatant was saved at −20°C. For each anti-Myc IP, 50 μl of packed Pierce anti-c-Myc agarose beads (Thermo Fisher Scientific) were prewashed and incubated with 1,000 μl of whole-cell lysate (1 to 2 μg/μl) for 2 h with rotation at 4°C. After six washes (5 min each) using a low-stringency buffer (containing 1 mM PMSF, 0.1% [vol/vol] Tween 20, and 0.1% [vol/vol] NP-40) at 4°C, IP bound proteins were released by adding 50 μl 2× reducing lane marker buffer (Thermo Fisher Scientific) and heated at 95°C for 5 min.

### Mass spectrometry sample preparation and data analysis.

Boiled IP samples were loaded onto a 4% to 12% SDS-PAGE gel (Thermo Fisher Scientific) and separated at 110 V for 8 min to get a 1.0-cm sample size. The sample lanes were cut and fixed at room temperature for 2 h and submitted to a mass spectrometry facility (SUMS) for standard LC-MS analysis according to standard procedures. Briefly, the gel was processed into small pieces and reduced with 5 mM DTT and 50 mM ammonium bicarbonate at 55°C for 30 min. The samples were then alkylated using 10 mM acrylamide in 50 mM ammonium bicarbonate for 30 min at room temperature. Overnight trypsin/LysC digestion (Promega) was performed in the presence of 0.02% ProteaseMAX (Promega) at 37°C. The samples were dried and reconstituted in 12.5 μl reconstitution buffer (2% acetonitrile with 0.1% formic acid), and 3 μl was used for the sample injection. LC-MS was performed on an Orbitrap Q-Exactive HFX mass spectrometer (Thermo Fisher Scientific) with liquid chromatography using a NanoACQUITY ultraperformance liquid chromatography (UPLC) system (Waters Corporation). The mass spectrometer was operated in a data-dependent fashion using high-energy collisional dissociation (HCD) fragmentation for MS/MS spectrum generation.

We performed three IP replicates for each sample using LC-MS analysis. Four samples (*Eh*Ago2-2 WT, the two *Eh*Ago2-2 mutants, and Luc control) were used to identify the core RISC components, and a total of 12 mass spectrometry data sets were generated. For data analysis, the mass spectrometry .RAW data files were first analyzed by Byonic v 2.14.27 (Protein Metrics) to identify peptides and search protein hits against the E. histolytica genome. Proteolysis was assumed to be tryptic allowing for N-ragged cleavage with up to two missed cleavage sites. Precursor and fragment mass accuracies were held within 12 ppm. Proteins were held to a false-discovery rate of 1% using standard approaches (36).

For each individual data set, all protein hits were first separated into ribosomal and nonribosomal hits; only nonribosomal hits were analyzed further in this study. Venn diagram analysis was used for three replicates for each sample to create a two-out-of-three list for nonribosomal proteins (i.e., hits are found in two of three replicates). This analysis was applied to all samples (*Eh*Ago2-2 WT, *Eh*Ago2-2^PAZ-mut^ and *Eh*Ago2-2^ΔNLS-DR^ mutants, and Luc control). We then compared Ago (WT or mutants) against the Luc control: protein hits not in the control were considered exclusive, and overlapping hits found in both were further analyzed for enrichment. The average spectral counts were used for enrichment analysis: protein hits with a calculated value in the Ago samples greater than the control (≥3) were considered enriched. Thus, two lists of protein hits were generated for each of the three Ago samples (WT and two mutants) as exclusive and enriched lists.

The Byonic output file ranked its protein hits by the log base 10 of the *P* value, known as the log *P* value. We used the log *P* value to rank protein hits into high-, moderate-, and low-confidence levels. Protein hits with a log *P* value greater than the top decoy (≥2) were considered high-confidence hits (confidence level, >99%); protein hits in the range of 0.7 to 2 were considered moderate-confidence hits (confidence levels between 80% and 99%); the rest that did not meet these two criteria were considered low-confidence hits.

For *Eh*Ago2-2 RISC confirmation, we performed mass spectrometry analysis for two *Eh*RdRPs and five protein hits selected from *Eh*Ago2-2 RISC. All seven proteins were Myc tagged and overexpressed in parasites. We performed anti-Myc IP from lysates prepared from these cell lines. The mass spectrometry runs for the *Eh*RdRP data sets were performed in triplicates, and data analysis was similar to the Ago RISC analysis. For the five selected RISC hits, mass spectrometry analysis was performed in duplicates. The criteria for selection included hits in both replicate data sets, and only exclusive hits after comparison with the Luc control were selected. The exclusive hits for each tagged protein sample were compared with the *Eh*Ago2-2 RISC hits for the overlap.

### Western blot analysis.

A standard Western blotting technique was performed. Briefly, the membranes were blocked using phosphate-buffered saline (PBS) containing 1% casein (Bio-Rad) and then probed with a 1:1,000 dilution of a primary antibody overnight at 4°C. The appropriate secondary antibody was used at a 1:10,000 dilution and incubated at room temperature for 1 h. Signal was detected with ECL+ (GE Healthcare), and the film was developed by a film processor (GE Healthcare). The antibodies used for Western blotting were mouse anti-Myc (Cell Signaling), rabbit anti-actin (Cell Signaling), rabbit anti-GST (Cell Signaling) antibodies and the custom-made *Eh*Ago2-2 polyclonal antibodies in rabbit (28).

### Immunofluorescence assay.

We followed the same protocol used in references 30 and 31. Briefly, parasites were fixed using methanol-acetone (1:1) and incubated with 1:250 mouse anti-Myc antibody overnight at 4°C. After washing with PBS three times, the slides were incubated with 1:1,000 Alexa 488-conjugated goat anti-mouse antibody for 1 h. The samples were mounted using Vectashield (Vector Laboratories, Inc.) and sealed with a cover slip. Images were collected using a Leica CTR6000 microscope using a BD CARVII confocal unit.

### Expression and purification of recombinant two NAP proteins.

For GST-tagged proteins, plasmids pGEX-NAP07 and pGEX-NAP12 were transformed in Escherichia coli BL21(DE3), and the two recombinant proteins (GST-NAP07 and GST-NAP12) were purified using glutathione beads (GE Healthcare). Briefly, overnight cultures were diluted 1:20 into LB and incubated at 37°C until the cell density reached an optical density at 600 nm (OD_600_) of 0.6 to 0.7. The cultures were induced with 0.5 mM IPTG for 2 to 4 h at room temperature, and cells were pelleted and stored at −20°C. To prepare the lysates, the frozen pellets were resuspended in GST lysis buffer (10 mM Tris [pH 7.5], 150 mM NaCl, 1 mM EDTA) plus 100 μg/ml lysozyme and incubated on ice for 30 min. Following incubation, 1.0% Triton X-100, 1 mM PMSF, and 1× Halt protease inhibitor cocktail were added, and the cells were lysed by sonication. After centrifugation at 14,000 rpm for 10 min at 4°C, the supernatants were saved at −20°C. For purification, glutathione beads were equilibrated with GST lysis buffer, and cell lysates were added. The samples were rotated at 4°C for 1 h to allow GST-tagged proteins to bind the glutathione resin. The beads were washed three times (5 min each time) with GST-wash buffer (50 mM Tris-HCl [pH 7.5], 350 mM NaCl, 1 mM PMSF). The bound proteins were eluted with elution buffer (20 mM reduced glutathione, 50 mM Tris-HCl [pH 8.0], 10 mM MgCl_2_, 1 mM PMSF, 1× Halt protease inhibitor cocktail). Purified protein samples were dialyzed overnight at 4°C in dialysis buffer (5 mM HEPES [pH 7.6], 1 mM DTT, 0.2 mM PMSF, 1 mM EDTA, 10% glycerol) with two changes to remove glutathione.

For His-tagged proteins, plasmids pET-NAP07 and pET-NAP12 were transformed into E. coli BL21(DE3), and the two His-tagged proteins (His-NAP07 and His-NAP12) were purified using HisPur nickel-nitrilotriacetic acid (Ni-NTA) resin (Thermo Fisher Scientific). Similar to the process described above, E. coli was cultured until an OD_600_ 0.6 to 0.7 and induced with 0.5 mM IPTG for 2 to 4 h at room temperature. The cells were pelleted and stored at −20°C. To prepare the lysates, the frozen pellets were resuspended in His-lysis buffer (PBS containing 10 mM imidazole) plus 100 μg/ml lysozyme and incubated on ice for 30 min. Then, 0.5% NP-40, 1 mM PMSF, and 1× Halt protease inhibitor cocktail were added, and the cells were lysed by sonication. The lysates were centrifuged, and the supernatants were stored at −20°C. For purification, Ni-NTA resin was equilibrated with His-lysis buffer, and the lysates were added. The samples were rotated at 4°C for 1 h to allow His-tagged proteins to bind the Ni-NTA resin. The beads were washed three times (5 min each) with His-wash buffer (PBS containing 35 mM imidazole and 1 mM PMSF). The bound proteins were eluted with His-elution buffer (PBS containing 250 mM imidazole, 1 mM PMSF, and 1× Halt protease inhibitor cocktail). Purified protein samples were dialyzed overnight at 4°C with two changes to remove imidazole.

### GST pulldown assay.

We used GST-tagged proteins to serve as “bait” for capturing putative His- or Myc-tagged “prey” binding partners. All bacterial lysates (GST, GST-NAP07, GST-NAP12, His-NAP07, His-NAP12, and His-ERM-BP) were made according to the detailed methods described in “Expression and purification of recombinant two NAP proteins.” For each sample, 25 μl of equilibrated glutathione beads was used, and 750 μl bait lysate was added to immobilize bait proteins. The sample mixture was rotated at 4°C for 1 h, and the beads were washed three times with 700 μl GST-wash buffer (see “Expression and purification of recombinant two NAP proteins”). We added 750 μl bacterial or amebic lysates containing prey proteins to the beads. The samples were rotated at room temperature for 15 min followed by a 1-h rotation at 4°C. The bound complexes were then washed again three times with GST-wash buffer and eluted with 50 μl GST-elution buffer (see “Expression and purification of recombinant two NAP proteins”). The eluted samples were separated by SDS-PAGE and stained by Coomassie blue G-250.

### Sequence alignment and phylogenetic analysis.

The genome database at https://amoebadb.org/amoeba/app was used. We searched nucleosome assembly protein domains in the genome and retrieved sequences for alignment analysis using Clustal Omega tool (www.ebi.ac.uk/Tools/msa/clustalo/), which also shows a phylogenetic tree.
