# Supplementary figures and images for: RISC in Entamoeba histolytica: Identification of a Protein-Protein Interaction Network for the RNA Interference Pathway in a Deep-Branching Eukaryote
Source: mBio. 2021 Sep 7;12(5):e01540-21. doi: 10.1128/mBio.01540-21 (PMC8546589; doi:10.1128/mBio.01540-21)

# Suppl. Fig. 1

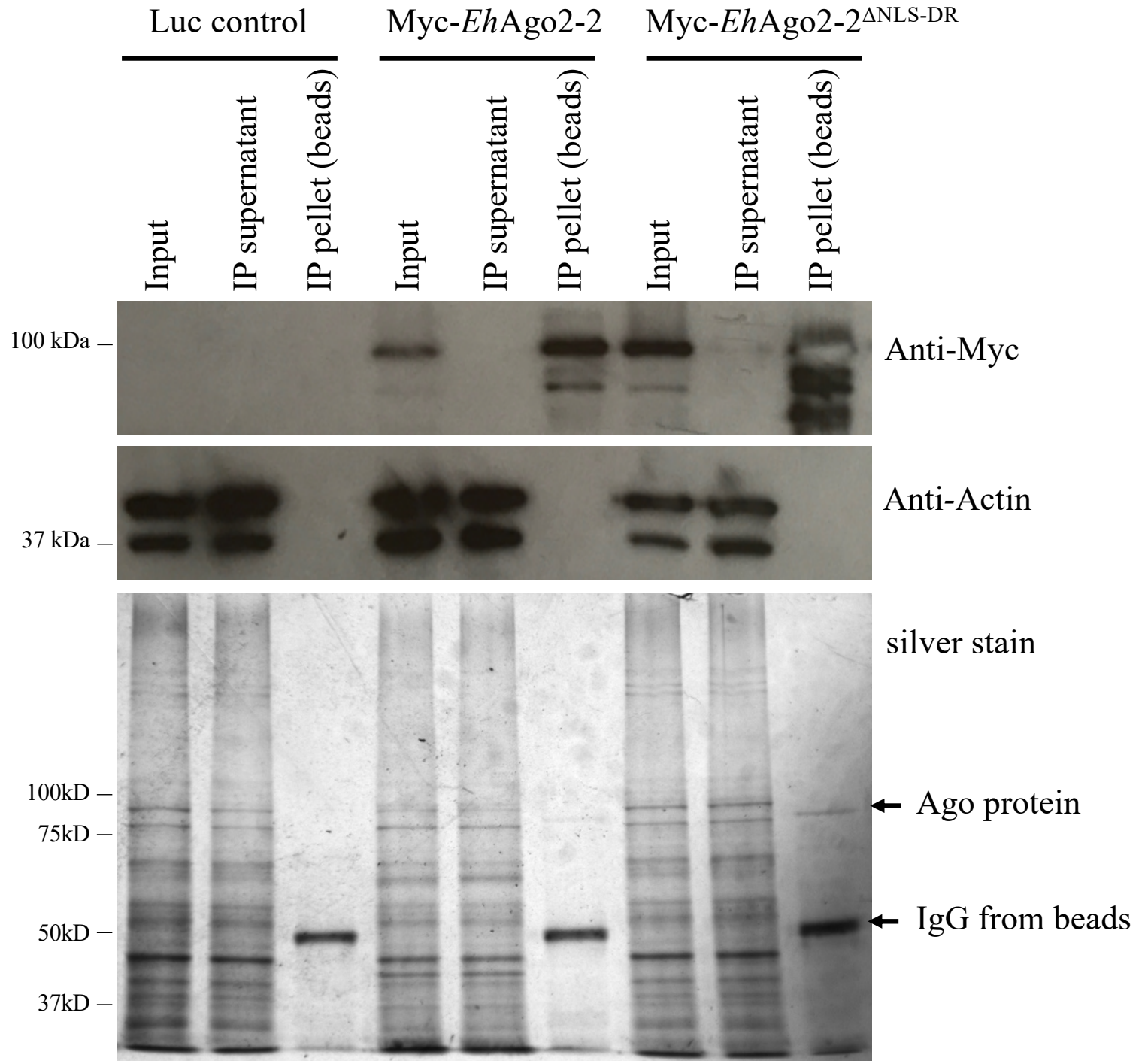

Supplement: FIG S1 [file mbio.01540-21-sf001.pdf]

**Suppl. Fig. 2A**

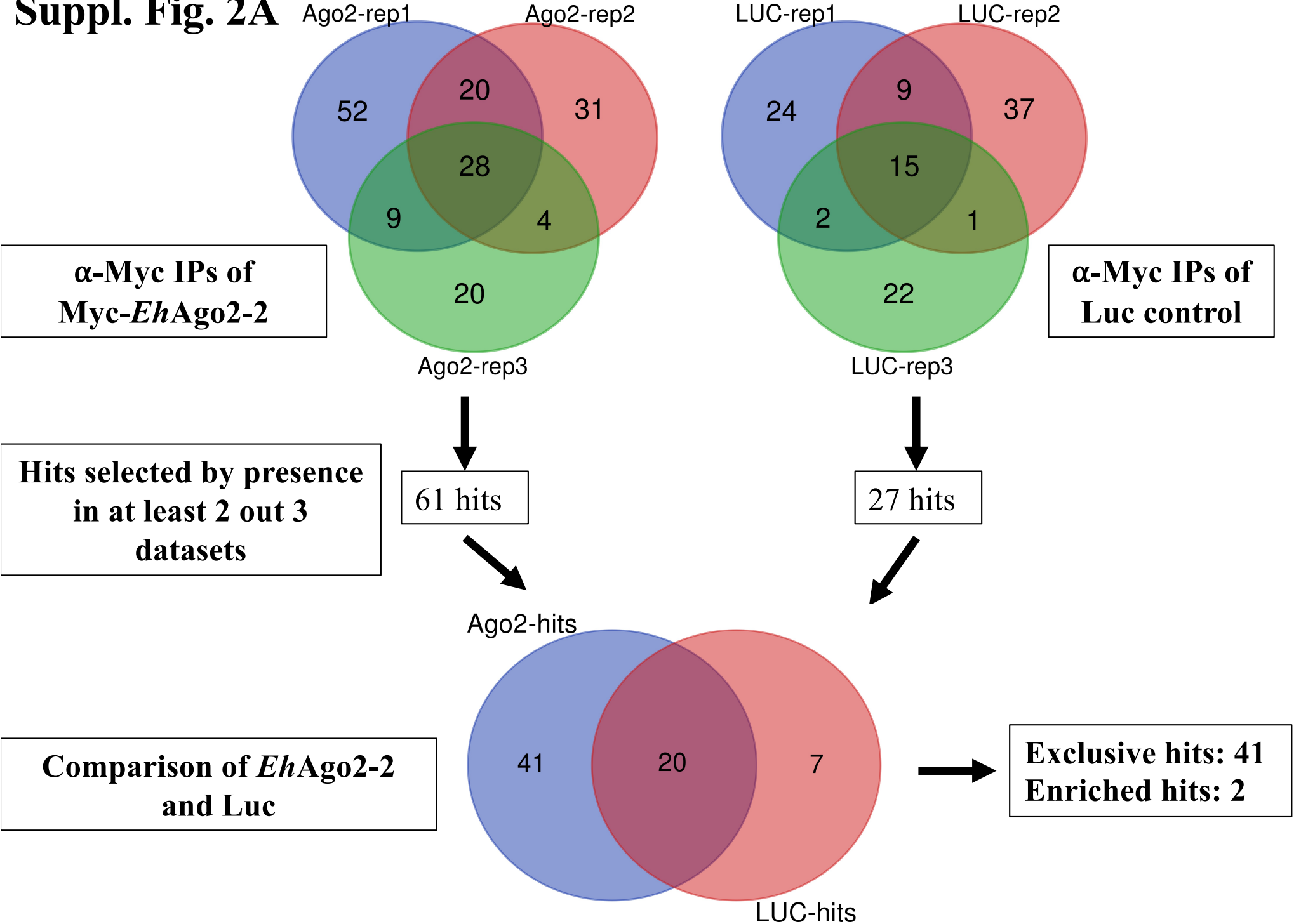

**Suppl. Fig. 2B**

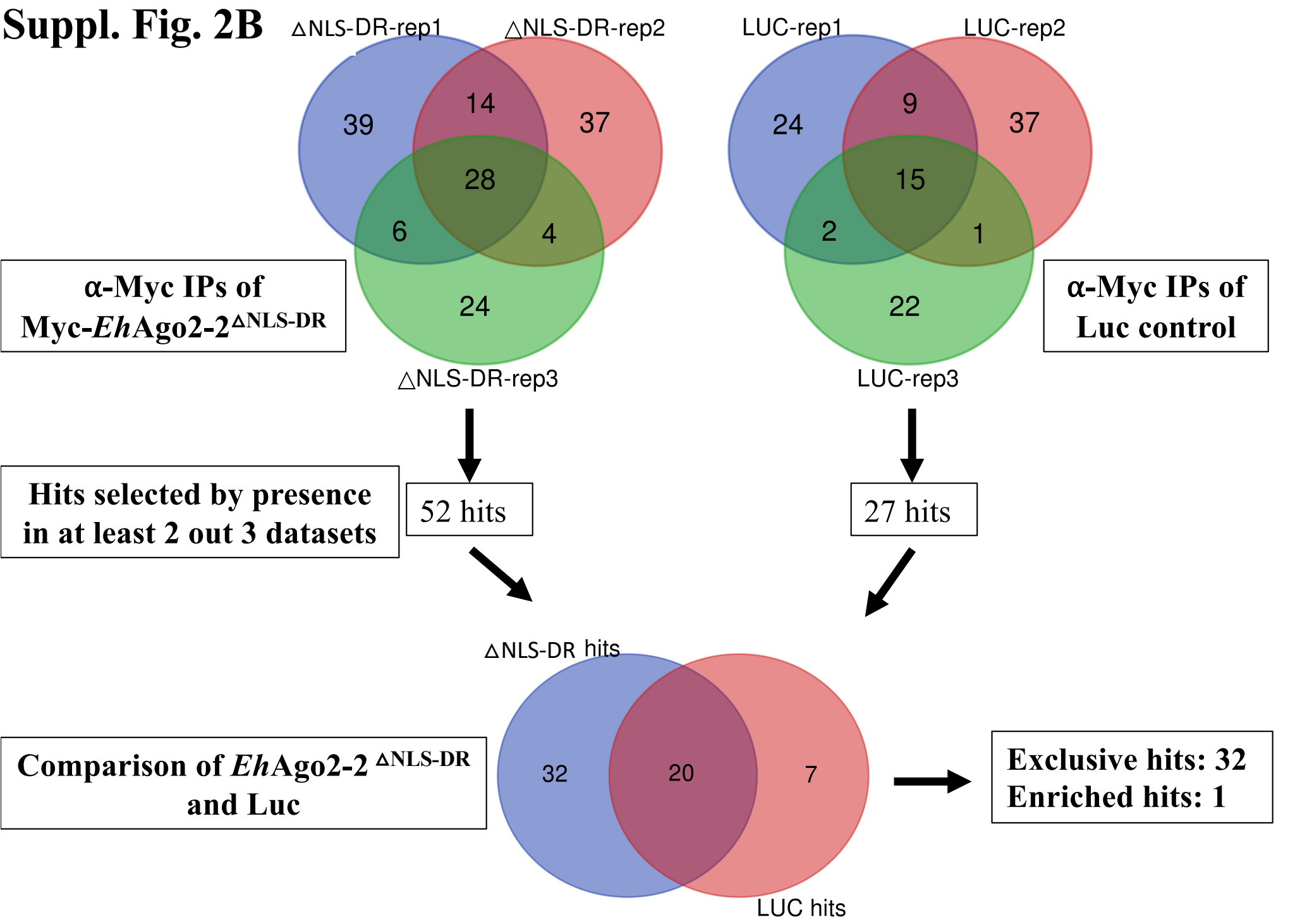

# Suppl. Fig. 2C

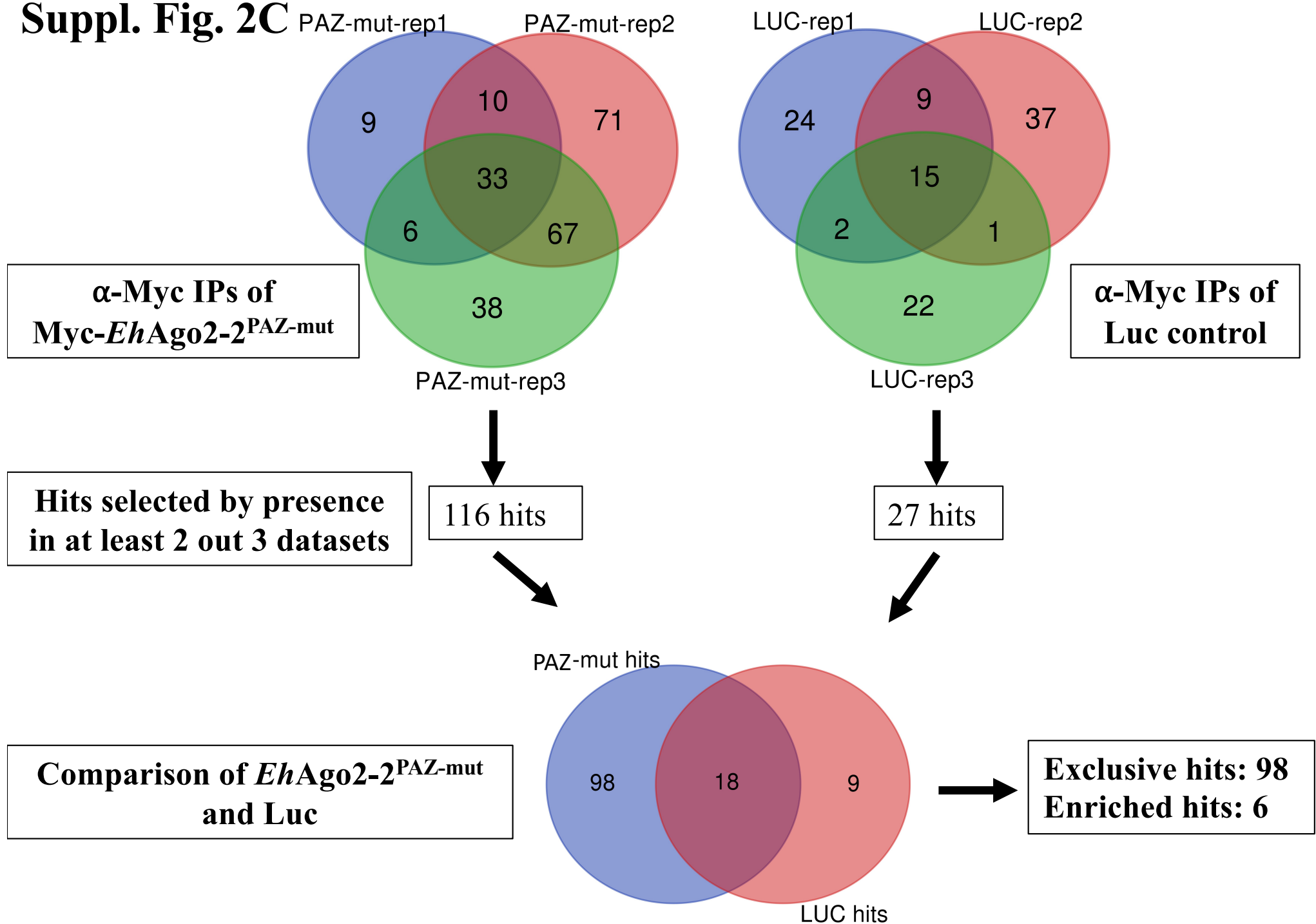

Supplement: FIG S2 [file mbio.01540-21-sf002.pdf]

Suppl. Fig. 4A

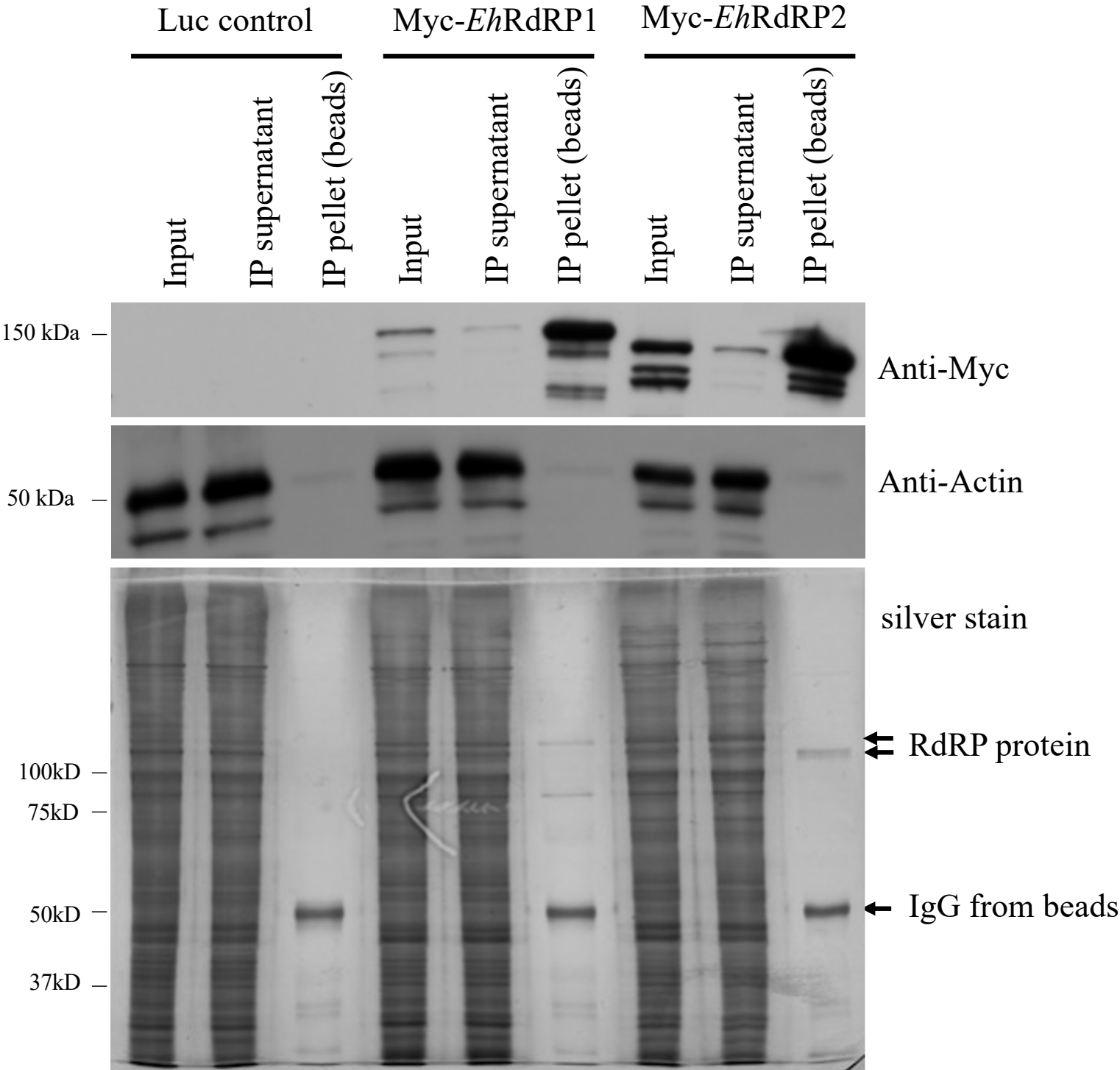

**Suppl. Fig. 4B**

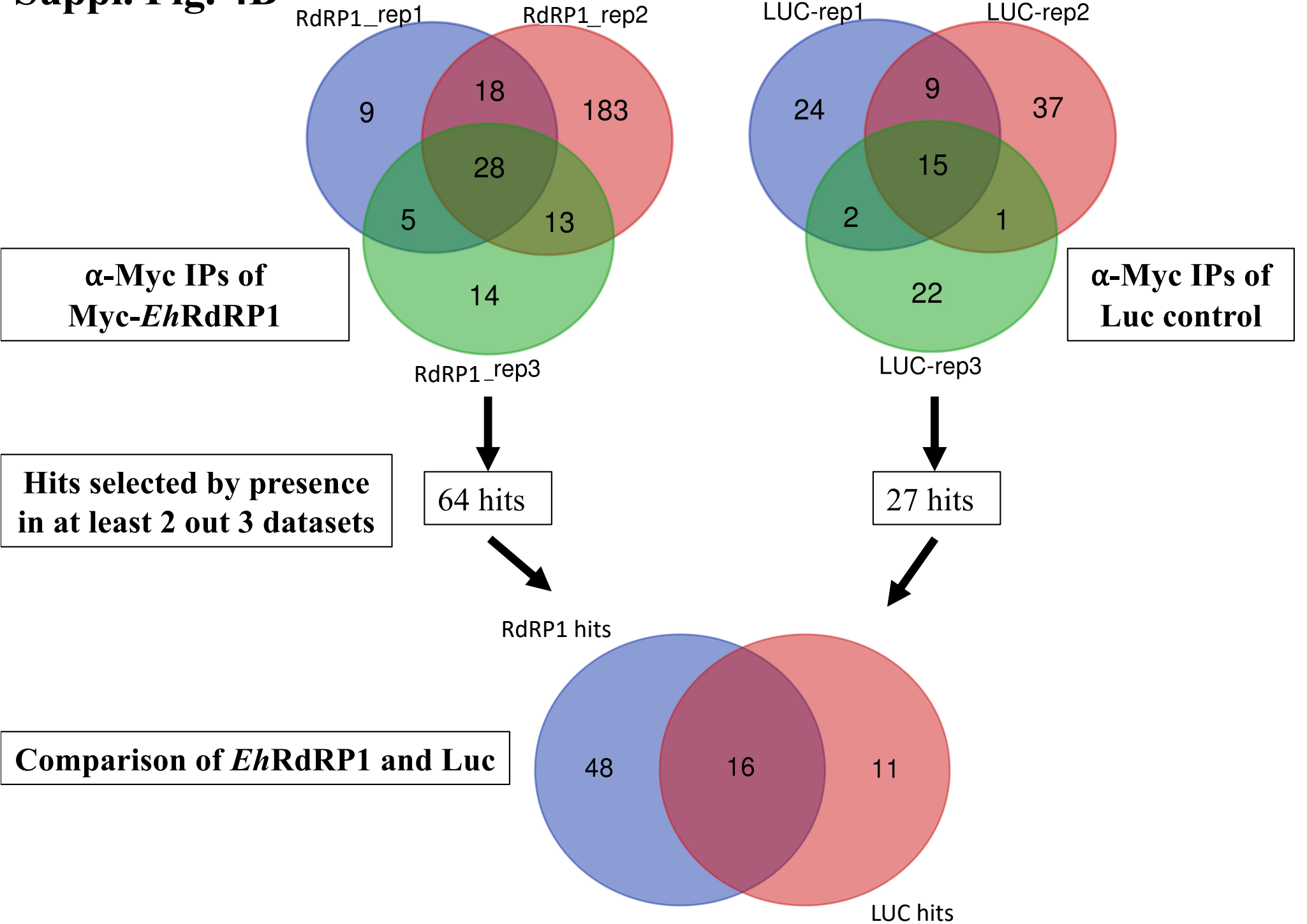

**Suppl. Fig. 4C**

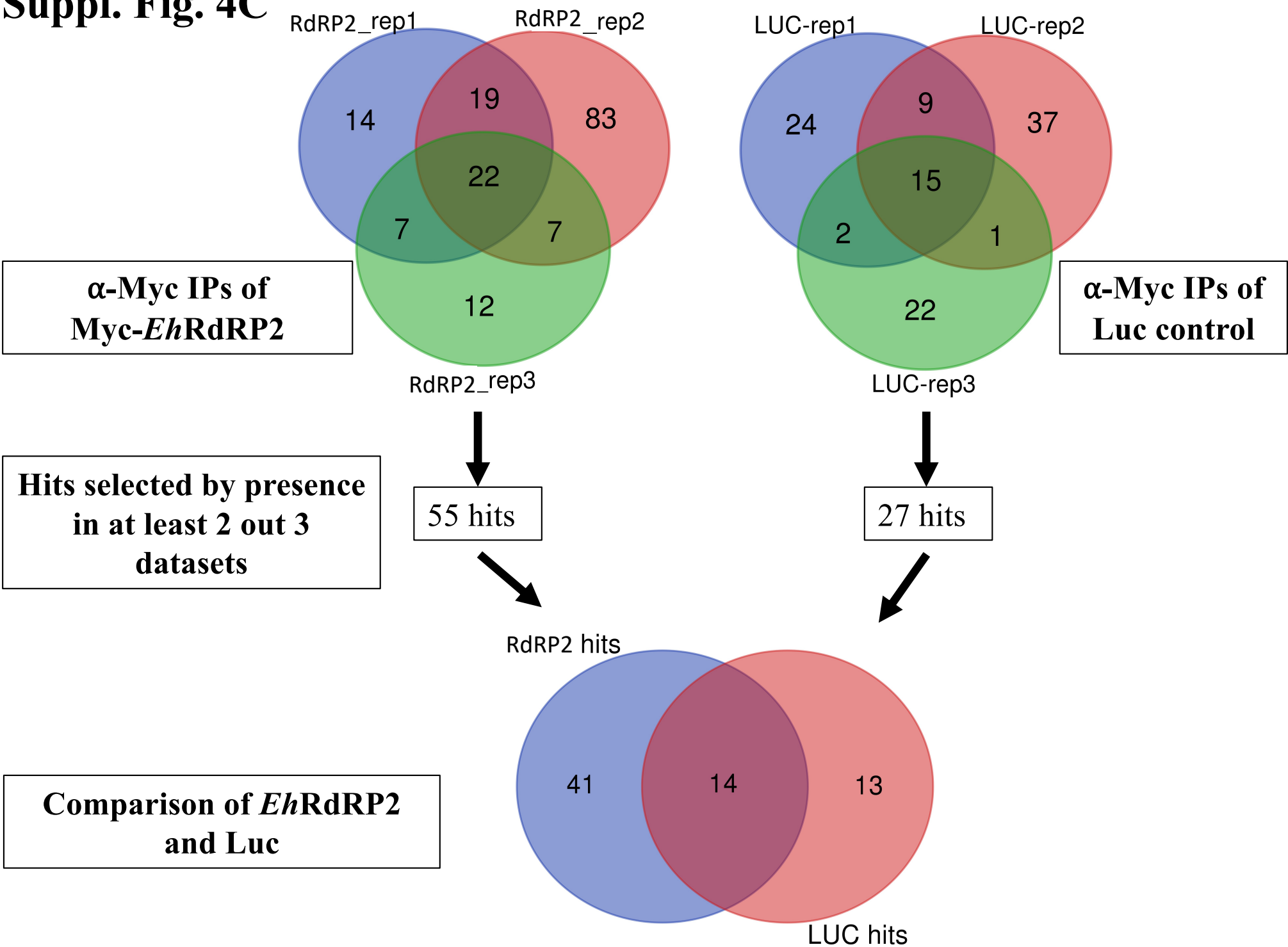

Supplement: FIG S4 [file mbio.01540-21-sf004.pdf]

Suppl. Fig. 5

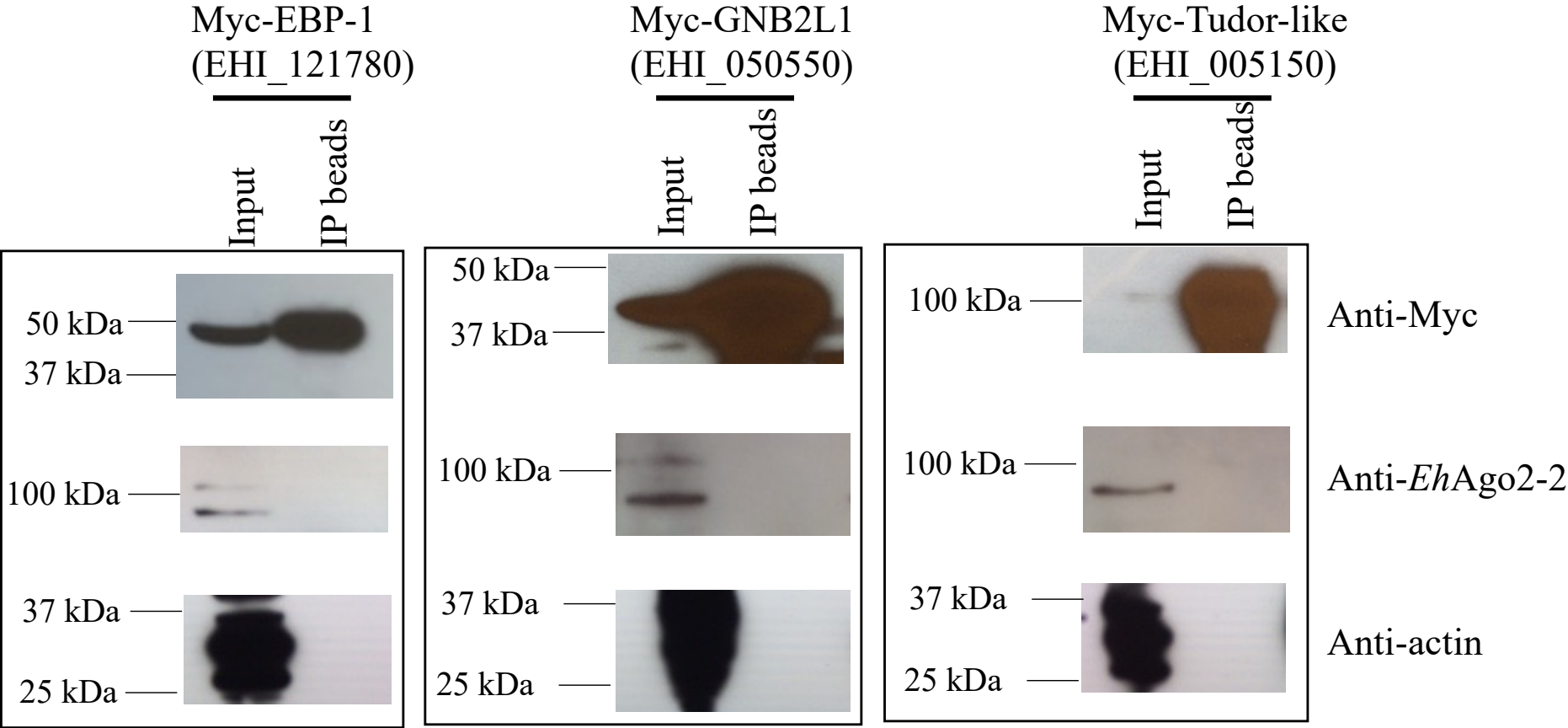

Supplement: FIG S5 [file mbio.01540-21-sf005.pdf]

## Suppl. Fig. 7A

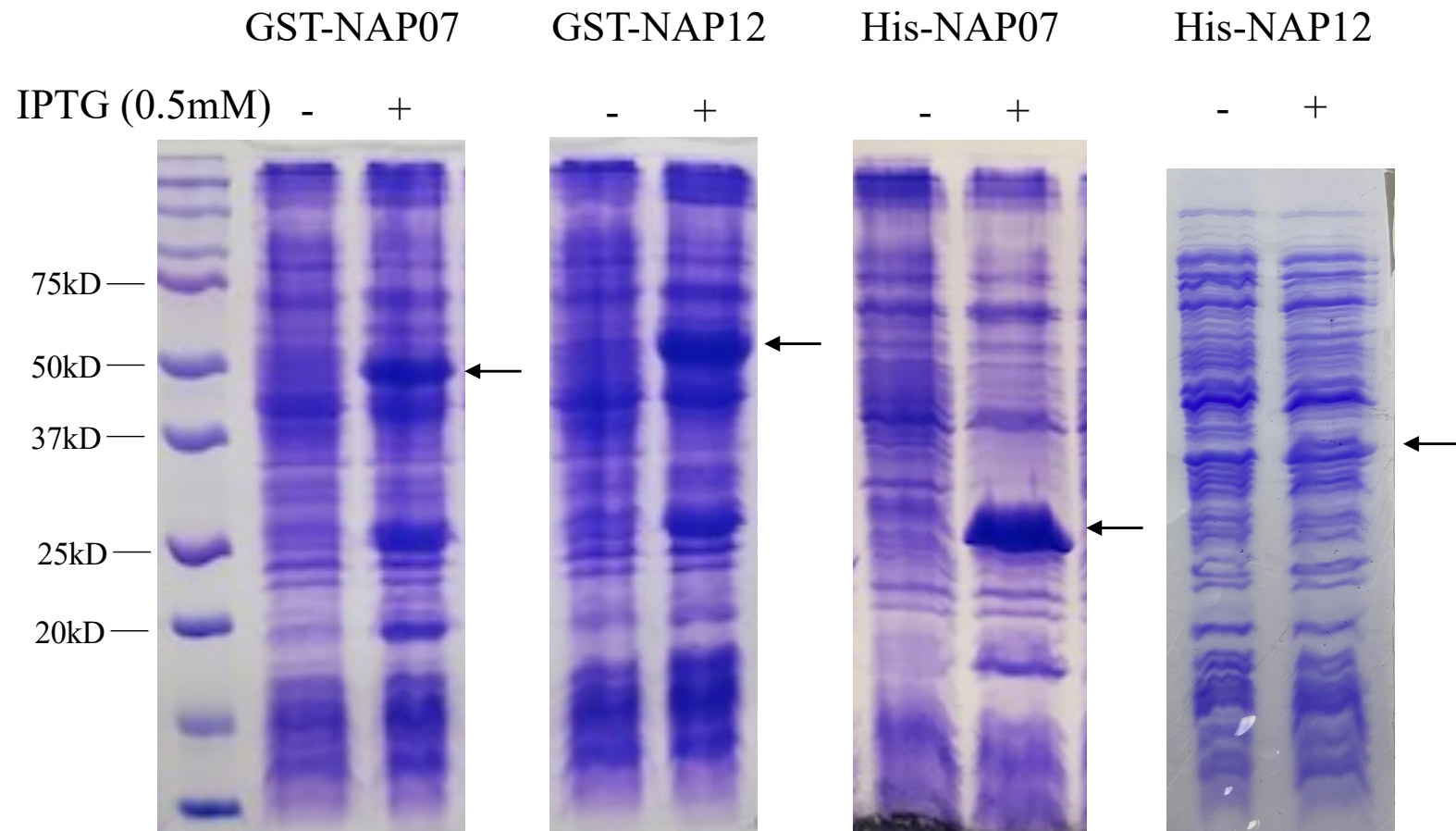

Suppl. Fig. 7B

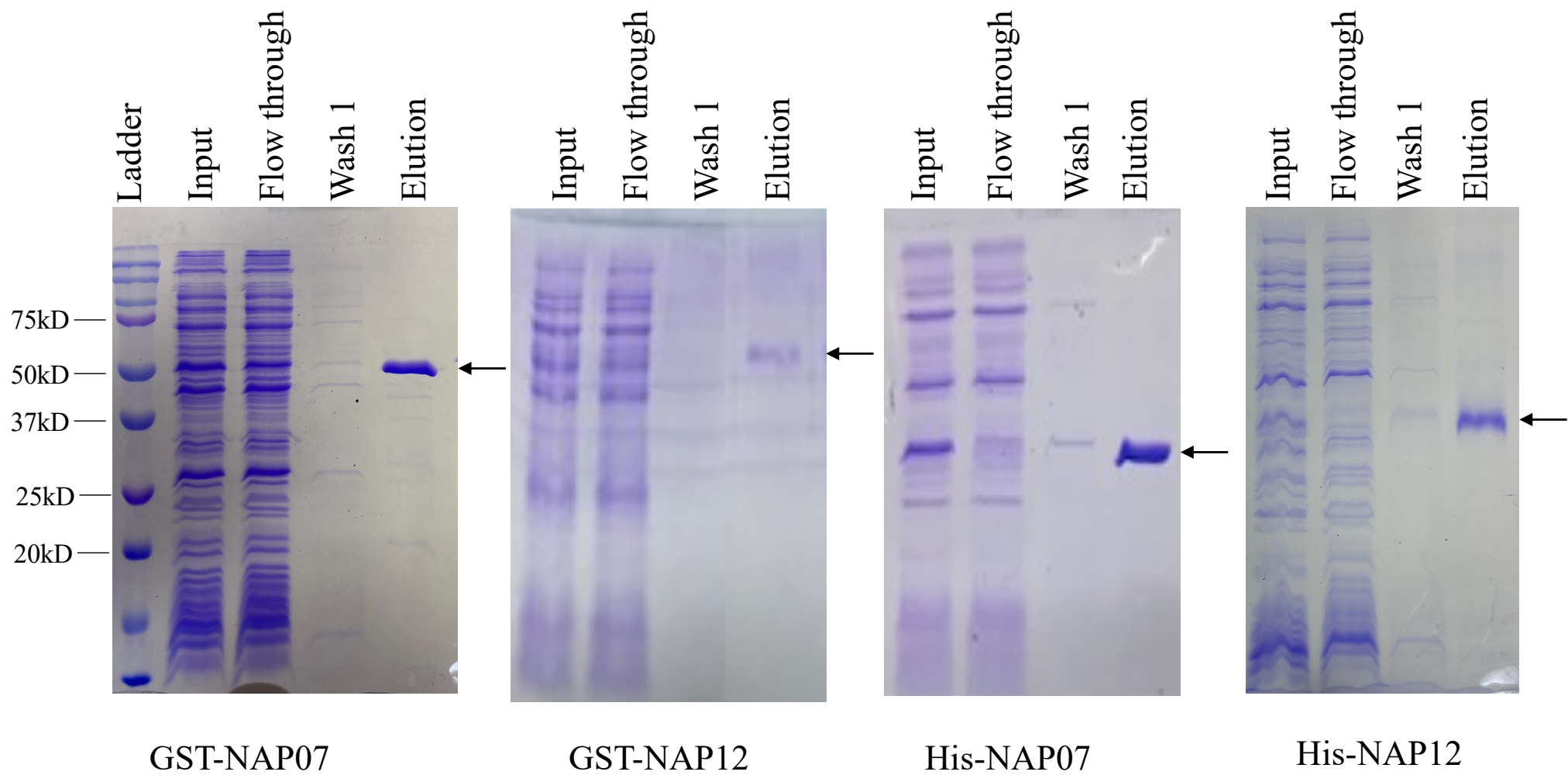

Suppl. Fig. 7C

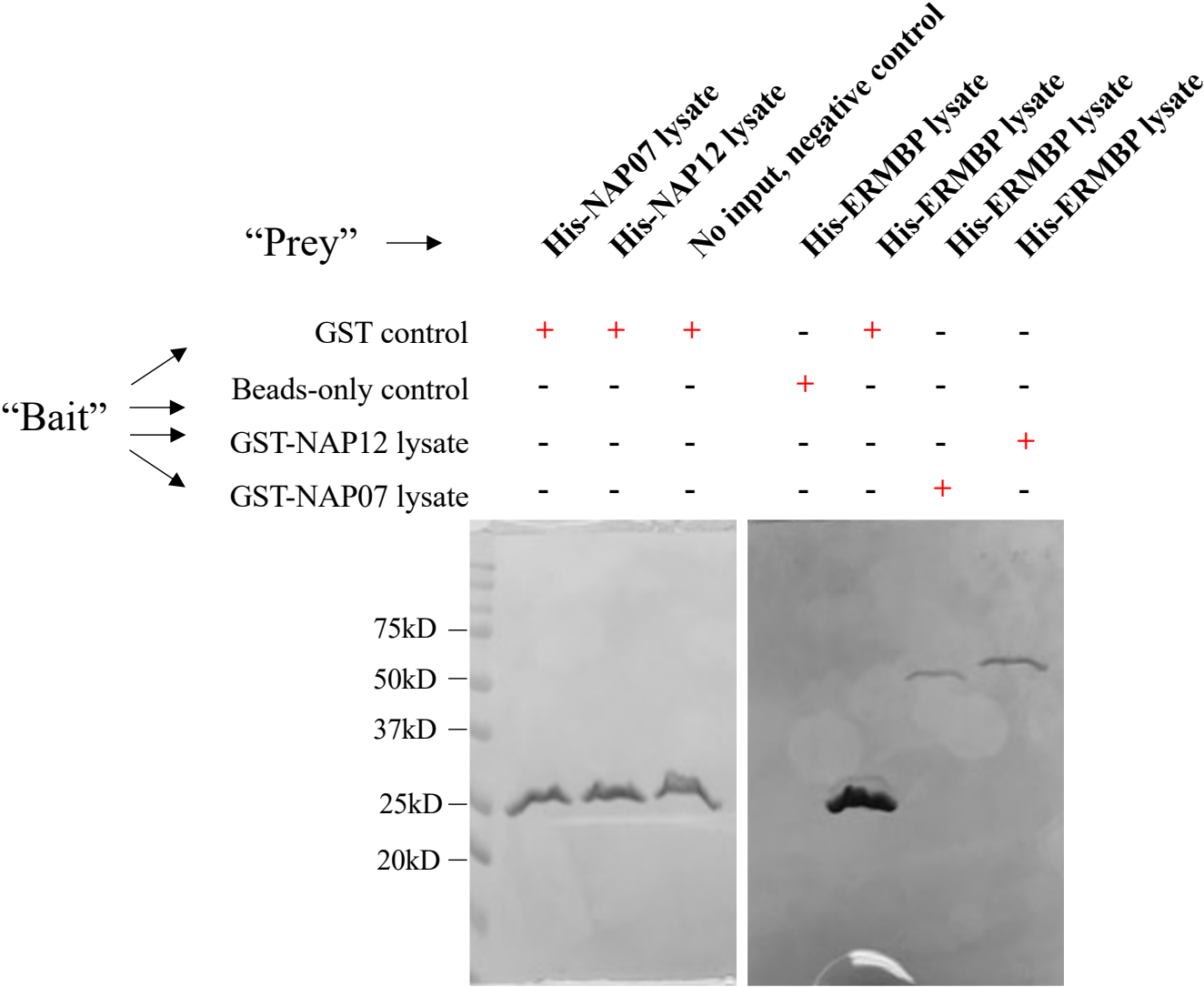

Supplement: FIG S7 [file mbio.01540-21-sf007.pdf]
