# Supplementary material for: RISC in Entamoeba histolytica: Identification of a Protein-Protein Interaction Network for the RNA Interference Pathway in a Deep-Branching Eukaryote
Source: mBio. 2021 Sep 7;12(5):e01540-21. doi: 10.1128/mBio.01540-21 (PMC8546589; doi:10.1128/mBio.01540-21)
Supplement: FIG S3 [file mbio.01540-21-sf003.pdf]

Suppl. Fig. 3A

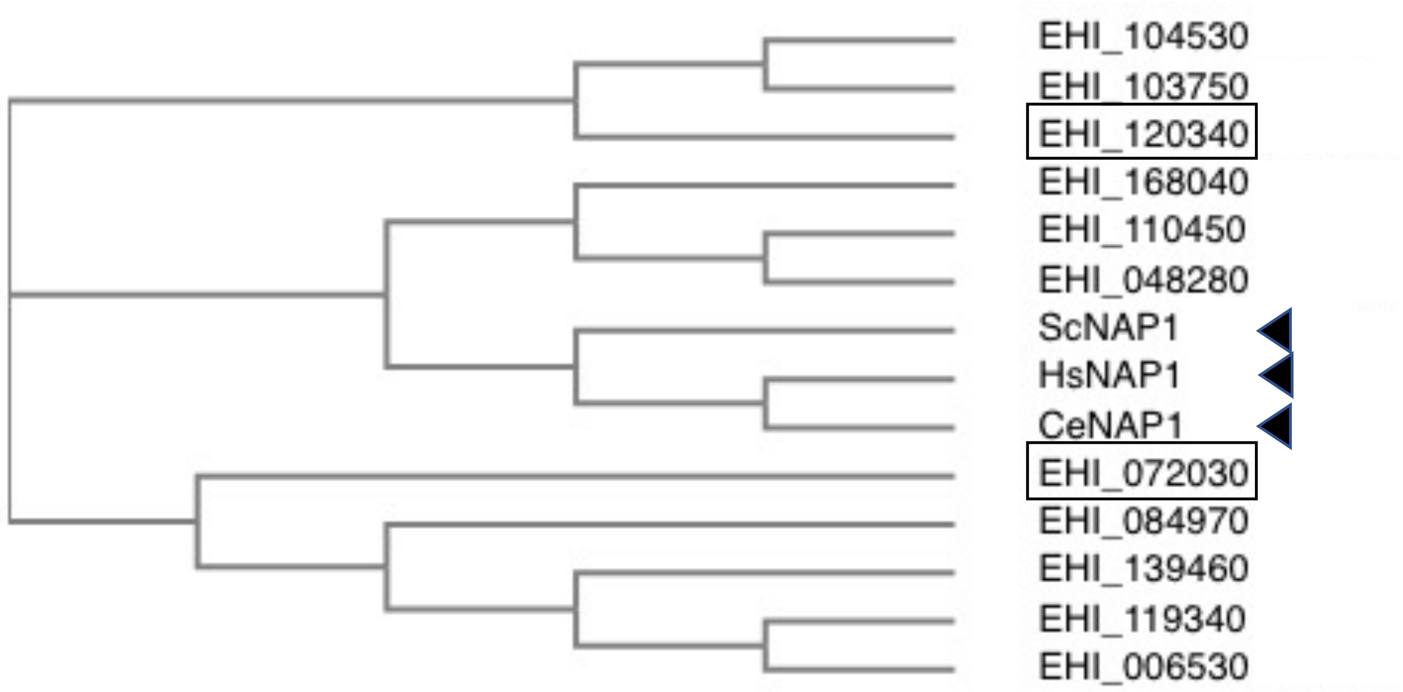
EHI_104530
EHI_103750
EHI_120340
EHI_168040
EHI_110450
EHI_048280
ScNAP1
HsNAP1
CeNAP1
EHI_072030
EHI_084970
EHI_139460
EHI_119340
EHI_006530

Suppl. Fig. 3B

```
NAP07       ------------------------------------------------------------      0
ceNAP1      ------------------------------------------------------------      0
yNAP1       MSDPIRTKPKSSMQIDNAPTPHNTPASVLNPSYLKNGNPVRAQAQEQDDKIGTINEEDIL      60

NAP07       ------MSYQ--------------SSQLPEDESINRKLLQETNRSLKIAQA--ELTVKKV      38
ceNAP1      ------MADQEHIDAGLLSTNFDMIQALPLN--VKQRVCALKNLQMKTIQIESDFYKRVH      52
yNAP1       ANQPLLLQSIQDRLGSLVGQDSGYVGGLPKN--VKEKLLSLKTLQSELFEVEKEFQVEMF      118
                  :                    ** :  ::.::   .. . :  :   ::  .

NAP07       YKTMEYEKMMKILNERRKDVVVGLSDQKSEESQQSKPIK----N--------DDVTSPKP      86
ceNAP1      ELEIEFEGKFKSTFDQRKAIVAGEVEPTKEQIDTP--ILEGLEGDQLA----ELYKAAEA      106
yNAP1       ELENKFLQKYKPIWEQRSRIISGQEQPKPEQIAKGQEIVESLNETELLVDEEEKAQNDSE      178
                ::    *   ::*. :: *  : . *:      *              :     .

NAP07       QINNKGVPFFWIRALNAVSLFLSYNTVEEDLVALSYLNDIKITTLTPSFDMKSLTIKMGK      146
ceNAP1      DPSAKGIKDFWLTALRTHDLVAE-AIEEHDVPILSYLTDVTTAAS-KD--------PAGF      156
yNAP1       EEQVKGIPSFWLTALENLPIVCD-TITDRDAEVLEYLQDIGLEYLTDG--------RPGF      229
            : . **:  **: **.   :. .    :.*   *.** *:       .          *

NAP07       ELSFFF--DKNPYFTNDHFTIRMIYRANENGER---IGSTGRIKVITNGIDWKVNLLE--      199
ceNAP1      KIEFHF--ATNPYFKNQVLTKTYLLGFDPDAEAPLQFDGPHVIRAVGDTIEWEDGKNVTK      214
yNAP1       KLLFRFDSSANPFFTNDILCKTYFYQKELGYSGDFIYD-----HAEGCEISWKDNAHNVT      284
            :: * *    **:*.*: :    :   : . .     .     :.    *.*: .

NAP07       ----------------------INSSSFFNVFIQELVNEE----------------DYEI      221
ceNAP1      KAV--KKKQKKGANAGKFLTKTVKADSFFNFFEPPKSKDERNEDEDDEQAEEFLELDYEM      272
yNAP1       VDLEMRKQRNKTTKQVRTIEKITPIESFFNFFDPPKIQNEDQDEELEEDLEERLALDYSI      344
                                     .****.*     ::*                **.:

NAP07       LDSVFDNFNTKAIQYFYQFN----------------------------------------      241
ceNAP1      GQAIRDTIIPRAVLFYTGELQSDDMFDFPGEDGDD-------VSDFSDDEA---------      316
yNAP1       GEQLKDKLIPRAVDWFTGAALEFEFEEDEEEADEDEDEEEDDDHGLEDDDGESAEEQDDF      404
             : : *.:  :*: ::

NAP07       -------------      241
ceNAP1      -------------      316
yNAP1       AGRPEQAPECKQS      417
```

Suppl. Fig. 3C

```
CLUSTAL O(1.2.4) multiple sequence alignment

NAP12       ------------------------------------------------------------       0
ceNAP1      ------------------------------------------------------------       0
yNAP1       MSDPIRTKPKSSMQIDNAPTPHNTPASVLNPSYLKNGNPVRAQAQEQDDKIGTINEEDIL       60

NAP12       ----MSIHSIDKINKVILSKNIDEYIDIVGGEVNENLEELKKIDKEIGEKEIEYQLECLK       56
ceNAP1      ------MADQEHIDAGLLSTNF-DMIQALPLNVKQRVCALKNLQMKTIQIESDFYKRVHE       53
yNAP1       ANQPLLLQSIQDRLGSLVGQDS-GYVGGLPKNVKEKLLSLKTLQSELFEVEKEFQVEMFE       119
                  : . :.    ::. :    :  :  :*::.:  **.:: :  : * ::  .  :

NAP12       LEQESEKDIQEILKIRRAIINGTKHRTND-----------------------------G       86
ceNAP1      LEIEFEGKFKSTFDQRKAIVAGEVEPTKEQIDTP--ILEGLEGDQLA----ELYKAAEAD       107
yNAP1       LENKFLQKYKPIWEQRSRIISGQEQPKPEQIAKGQEIVESLNETELLVDEEEKAQNDSEE       179
            ** :   . :   . *  *: *  . . :

NAP12       IQECGISNFWIEVFNEVGLMGNLEVTKEEIDILQSLIDIEKHIKEIKRNEGKEEIYINIH       146
ceNAP1      PSAKGIKDFWLTALRTHDLVAEA-IEEHDVPILSYLTDVTTAAS-----KDPAG--FKIE       159
yNAP1       EQVKGIPSFWLTALENLPIVCDT-ITDRDAEVLEYLQDIGLEYLT----DGRPG--FKLL       232
             .  ** .**: .:.   :: :  : ..:  :*. * *:          ..     :::

NAP12       YTFLFKENQFLSNSIF----------------EFELEHCINRYGIIIEETRNYIPLSHFK       190
ceNAP1      FHF--ATNPYFKNQVLTKTYLLGFDPDAEAPLQFDGPHVIRAVGDT------------IE       205
yNAP1       FRFDSSANPFFTNDILCKTYFYQKELGYSGDFIYD-----HAEGCE------------IS       275
            : *    * ::.*.::                 ::     .  *              :.

NAP12       WKKQIKEKRMF---DLLENGGKNLHCYLHEALNNIDFGAIDMFFGINTQNLFLDSFSVSD       247
ceNAP1      WEDGKNVTKKAV--KKKQKKGANAGKFLTK------TVKADSFFNF------FEPPKSKD       251
yNAP1       WKDNAHNVTVDLEMRKQRNKTTKQVRTIEK------ITPIESFFNF------FDPPKIQN       323
            *:.  :           .:   :    : :          : **.:      ::  . .:

NAP12       DENDCSVDDG-----CVDYATH--------------------------------------       264
ceNAP1      ERNEDEDDEQAEEFLELDYEMGQAIRDTIIPRAVLFYTGELQSDDMFDFPGEDGDD----       307
yNAP1       EDQDEELEEDLEERLALDYSIGEQLKDKLIPRAVDWFTGAALEFEFEEDEEEADEDEDEE       383
            : :: . ::       :**

NAP12       ----------------------------------  264
ceNAP1      ---VSDFSDDEA----------------------  316
yNAP1       EDDDHGLEDDDGESAEEQDDFAGRPEQAPECKQS  417
```
