# Supplementary material for: RISC in Entamoeba histolytica: Identification of a Protein-Protein Interaction Network for the RNA Interference Pathway in a Deep-Branching Eukaryote
Source: mBio. 2021 Sep 7;12(5):e01540-21. doi: 10.1128/mBio.01540-21 (PMC8546589; doi:10.1128/mBio.01540-21)
Supplement: FIG S6 [file mbio.01540-21-sf006.pdf]

Suppl. Fig. 6A

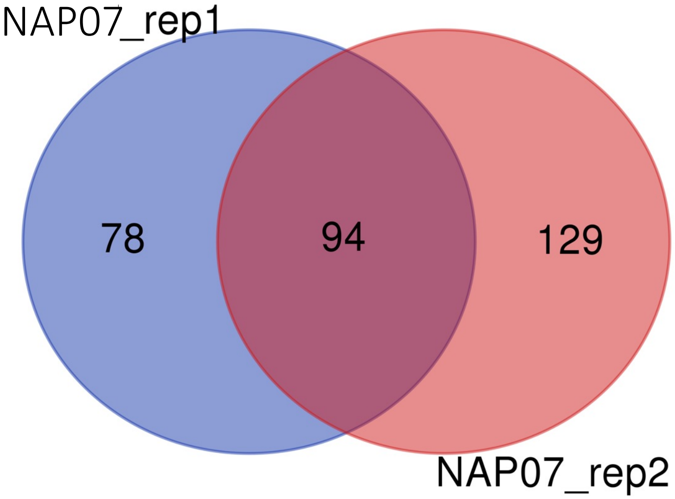

Hits in 2 out of 2 datasets

94 hits

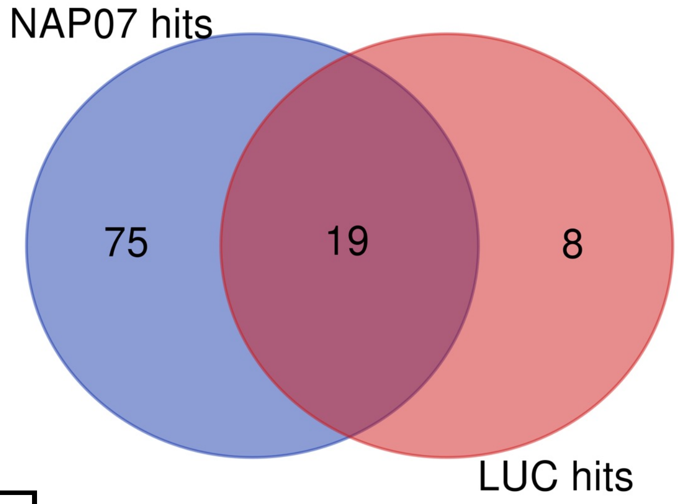

Exclusive hits: 75

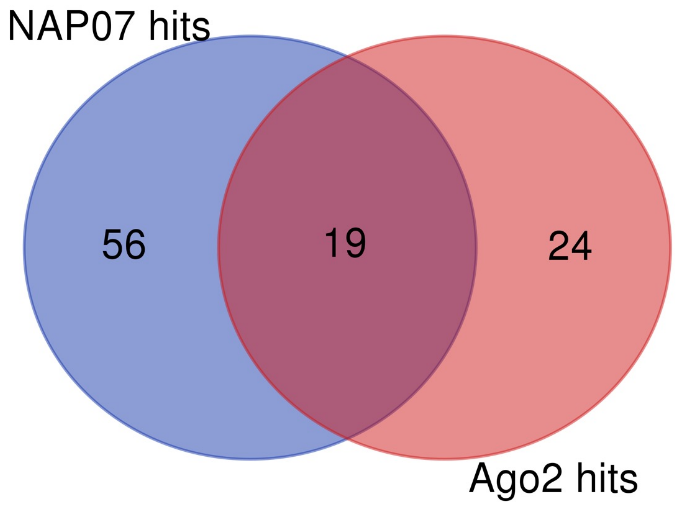

Myc-NAP07 IP	rep1 |Log Prob|	rep2 |Log Prob|	Annotation
EHI_072030	537.35	279.24	nucleosome assembly protein, putative
EHI_125650	6.95	112.84	PIWI, putative
EHI_120340	44.78	57.23	nucleosome assembly protein, putative
EHI_182520	57.13	49.18	DNAJ homolog subfamily A member 1, putative
EHI_121780	35.34	47.64	enhancer binding protein-1
EHI_151260	92.41	42.41	DnaJ family protein
EHI_199590	95.47	41.42	70 kDa heat shock protein, putative
EHI_005150	30.29	40.89	hypothetical protein, conserved
EHI_110400	10.51	24.07	guanine nucleotide-binding protein subunit beta 2-like 1, putative
EHI_128100	4.07	17.56	peptidyl-prolyl cis-trans isomerase, putative
EHI_094100	24.5	15.37	galactokinase, putative
EHI_100480	20.9	12.39	MIR domain protein
EHI_163540	7.82	8.67	estradiol 17-beta-dehydrogenase, putative
EHI_044970	11.2	7.5	malic enzyme, putative
EHI_050330	11.2	7.5	malic enzyme, putative
EHI_000610	13.45	6.89	nucleotide binding protein 2, putative
EHI_021560	4.17	5.42	thioredoxin, putative
EHI_060740	10.79	4.4	EF-hand calcium-binding domain containing protein
EHI_109860	1.71	2.78	chromatin-specific transcription elongation factor, putative

Suppl. Fig. 6B

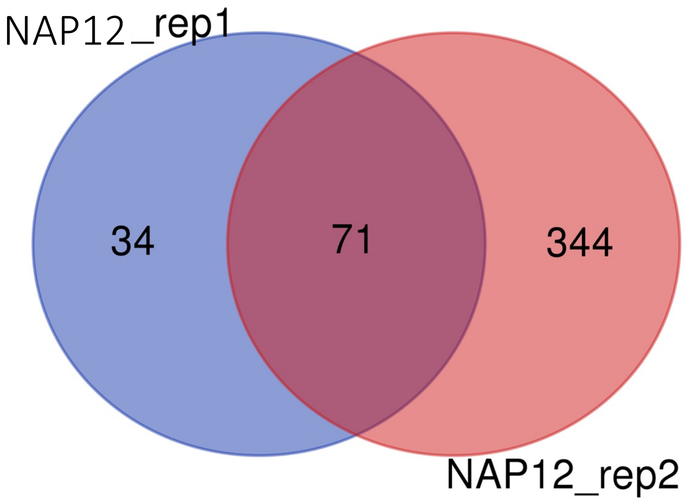

Hits in 2 out of 2 datasets

71 hits

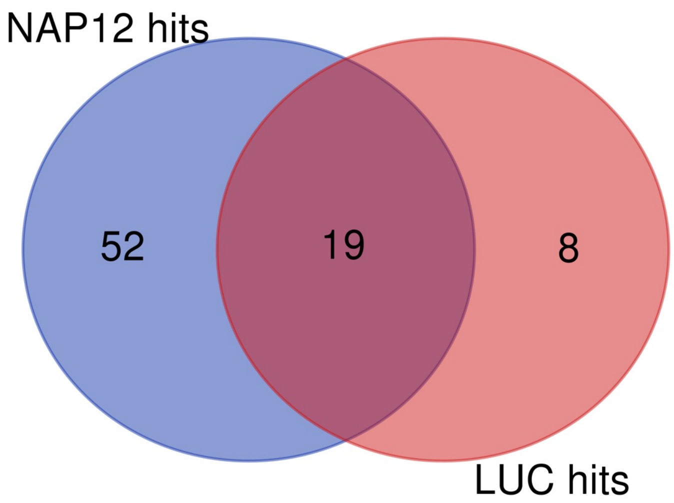

Exclusive hits: 52

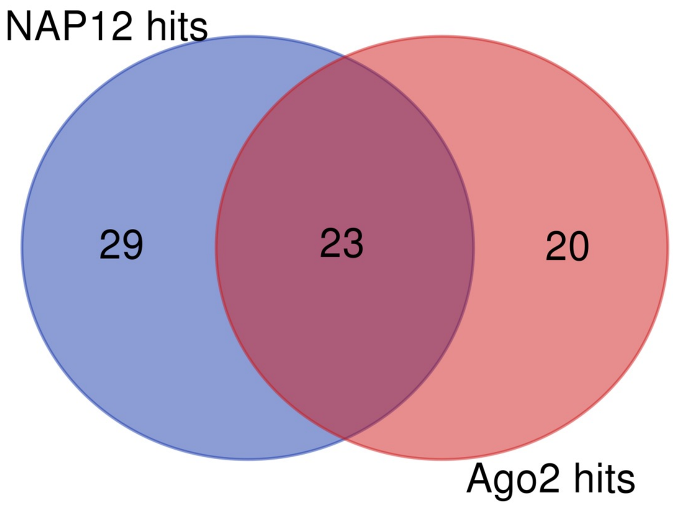

Myc-NAP12 IP	rep1 |Log Prob|	rep2 |Log Prob|	Annotation
EHI_072030	114.46	159.59	nucleosome assembly protein, putative
EHI_005150	91.17	334.09	hypothetical protein, conserved
EHI_120340	81.61	282.07	nucleosome assembly protein, putative
EHI_121780	45.73	151.2	enhancer binding protein-1
EHI_001420	40.19	65.54	peroxiredoxin
EHI_061980	40.19	65.54	peroxiredoxin
EHI_114010	40.19	0.82	peroxiredoxin
EHI_201250	40.19	0.81	peroxiredoxin
EHI_178570	28.83	74.04	chaperonin 1 60 kDa
EHI_110400	18.44	56.29	guanine nucleotide-binding protein subunit beta 2-like 1, putative
EHI_000610	8.58	21.2	nucleotide binding protein 2, putative
EHI_125650	6.47	440.6	PIWI, putative
EHI_044970	6.25	39.97	malic enzyme, putative
EHI_050330	6.25	39.97	malic enzyme, putative
EHI_021560	6.11	8.56	thioredoxin, putative
EHI_199590	5.22	42.64	70 kDa heat shock protein, putative
EHI_182520	4.86	40.95	DNAJ homolog subfamily A member 1, putative
EHI_151260	4.58	48.71	DnaJ family protein
EHI_141940	4.2	6.71	hypothetical protein
EHI_128100	4.02	19.85	peptidyl-prolyl cis-trans isomerase, putative
EHI_109860	3.73	4.83	chromatin-specific transcription elongation factor, putative
EHI_102170	0.52	23.04	elongation factor 1-alpha 1
EHI_052400	0.52	23.04	elongation factor 1-alpha 1

Suppl. Fig. 6C

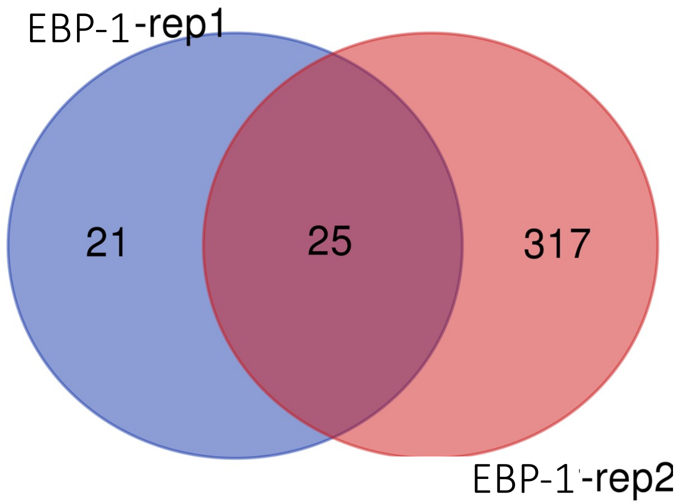

Hits in 2 out of 2 datasets

25 hits

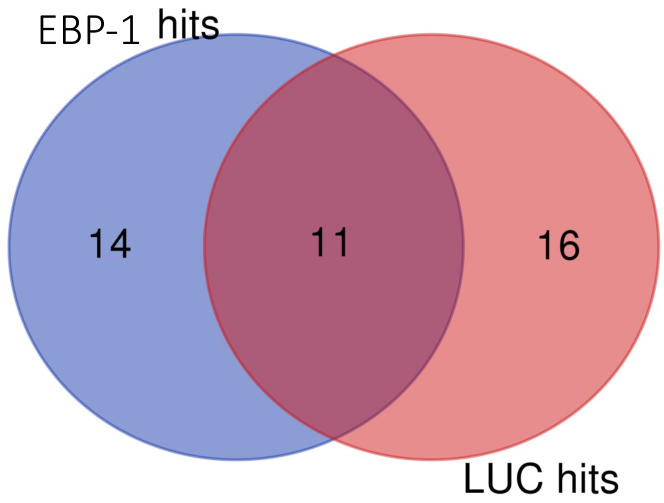

Exclusive hits: 14

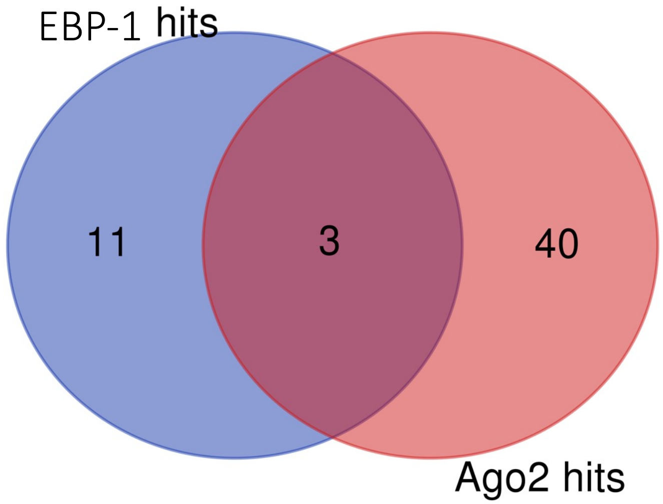

Myc-EBP-1 IP	rep1 |Log Prob|	rep2 |Log Prob|	Annotation
EHI_121780	8.07	1011.09	enhancer binding protein-1
EHI_005150	3.13	390.92	hypothetical protein, conserved
EHI_110400	5.77	223.84	guanine nucleotide-binding protein subunit beta 2-like 1, putative

Suppl. Fig. 6D

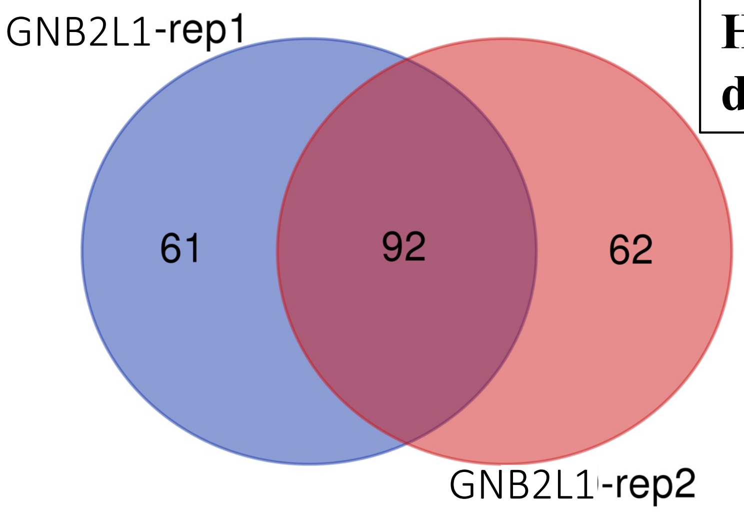

Hits in 2 out of 2 datasets

92 hits

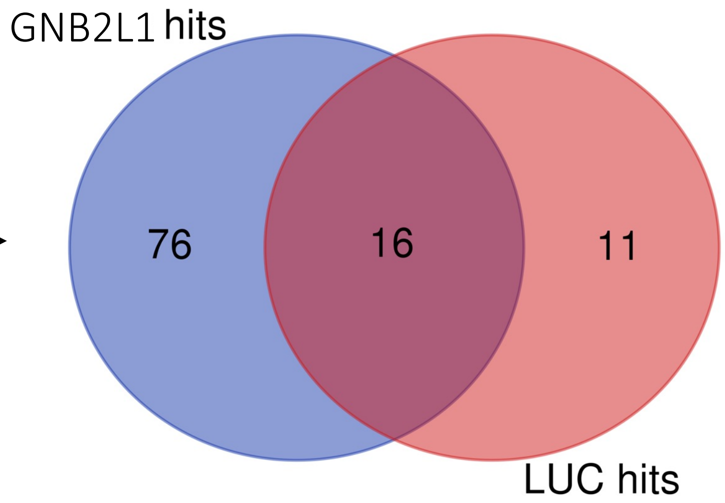

Exclusive hits: 76

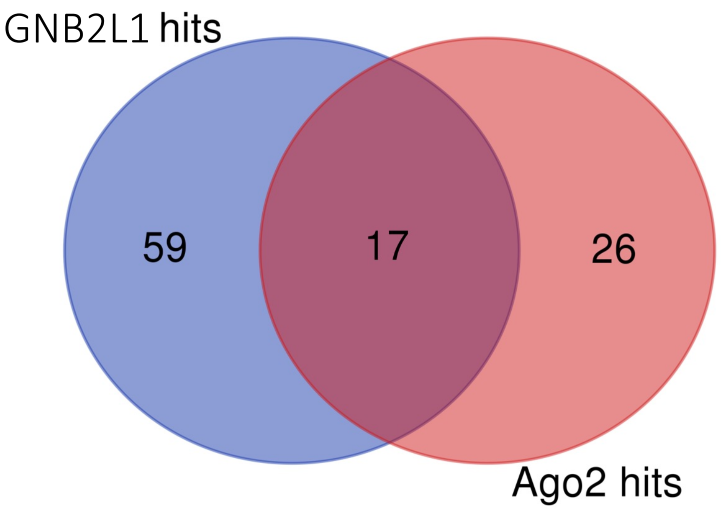

Myc-GNB2L1 IP	rep1	rep2	
	|Log Prob|	|Log Prob|	Annotation
EHI_050550	1320.44	736.45	guanine nucleotide-binding protein subunit beta 2-like 1, putative
EHI_005150	202.57	150.33	hypothetical protein, conserved
EHI_121780	153.24	110.13	enhancer binding protein-1
EHI_114010	62.33	37.29	peroxiredoxin
EHI_201250	62.33	37.29	peroxiredoxin
EHI_199590	11.21	12.44	70 kDa heat shock protein, putative
EHI_128100	19.02	11.17	peptidyl-prolyl cis-trans isomerase, putative
EHI_094100	24.84	8.31	galactokinase, putative
EHI_102170	21.3	7.74	elongation factor 1-alpha 1
EHI_052400	21.3	7.74	elongation factor 1-alpha 1
EHI_060740	5.55	7.63	EF-hand calcium-binding domain containing protein
EHI_201710	20.57	4.79	clathrin heavy chain, putative
EHI_119330	15.53	3.33	serine protease inhibitor, putative
EHI_050330	52.08	1.61	malic enzyme, putative
EHI_044970	52.08	1.61	malic enzyme, putative
EHI_141940	7.48	1.26	hypothetical protein
EHI_000610	14.09	0.92	nucleotide binding protein 2, putative

Suppl. Fig. 6E

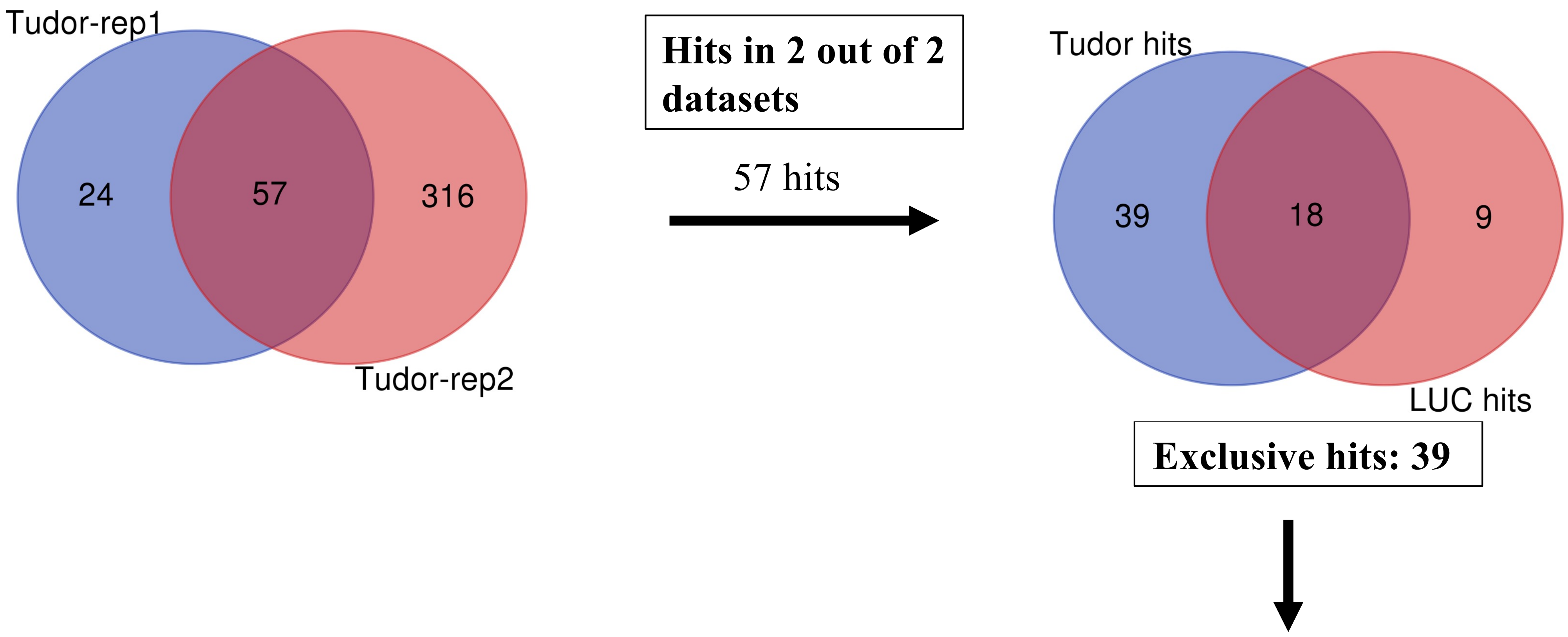

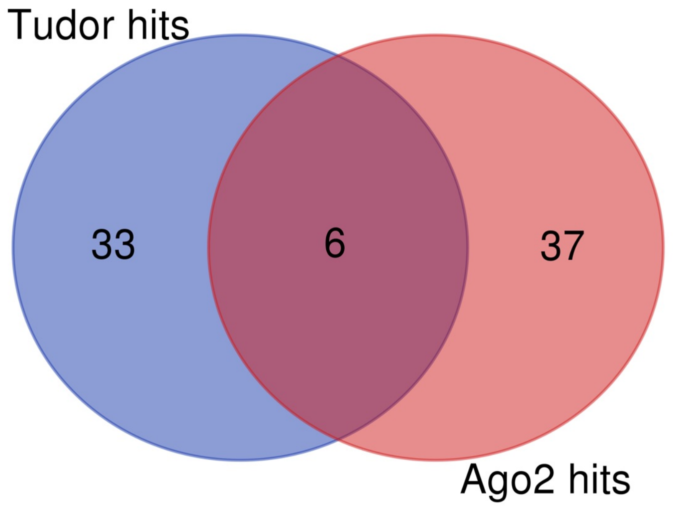

Myc-Tudor-like IP	rep1 |Log Prob|	rep2 |Log Prob|	Annotation
EHI_005150	160.94	2897.02	hypothetical protein, conserved
EHI_121780	11.52	248.15	enhancer binding protein-1
EHI_109860	4.94	215.8	chromatin-specific transcription elongation factor, putative
EHI_110400	4.54	136.51	guanine nucleotide-binding protein subunit beta 2-like 1, putative
EHI_114010	2.05	57.88	peroxiredoxin
EHI_201250	2.05	56.23	peroxiredoxin
